# Natural killer cells regulate eosinophilic inflammation in chronic rhinosinusitis

**DOI:** 10.1038/srep27615

**Published:** 2016-06-08

**Authors:** Ji Heui Kim, Go Eun Choi, Bong-Jae Lee, Seog Woon Kwon, Seung-Hyo Lee, Hun Sik Kim, Yong Ju Jang

**Affiliations:** 1 Department of Otolaryngology, Asan Medical Center, University of Ulsan College of Medicine, Seoul 138-736, Korea; 2 Department of Biomedical Sciences, University of Ulsan College of Medicine, Seoul 138-736, Korea; 3 Institute of Convergence Bio-Health, Dong-A University, Busan, Republic of Korea; 4 Department of Laboratory Medicine, University of Ulsan College of Medicine, Asan Medical Center, Seoul 138-736, Korea; 5 Graduate School of Medical Science and Engineering, Biomedical Research Center, KAIST Institute for the BioCentury, Korea Advanced Institute of Science and Technology, Daejeon 305-701, Korea; 6 Cellular Dysfunction Research Center, University of Ulsan College of Medicine, Seoul 138-736, Korea; 7 Department of Microbiology, University of Ulsan College of Medicine, Seoul 138-736, Korea

## Abstract

Eosinophils play a major pathologic role in the pathogenesis of diverse inflammatory diseases including chronic rhinosinusitis (CRS). Dysregulated production of prostaglandin (PG), particularly PGD_2_, is considered to be an important contributing factor to eosinophilic inflammation in CRS primarily through proinflammatory and chemotactic effects on eosinophils. Here, we provide evidence that PGD_2_ can promote eosinophilic inflammation through a suppression of Natural killer (NK) cell effector function and NK cell-mediated eosinophil regulation. Eosinophil apoptosis mediated by NK cells was significantly decreased in CRS patients compared with healthy controls. This decrease was associated with NK cell dysfunction and eosinophilic inflammation. Tissue eosinophils were positively correlated with blood eosinophils in CRS patients. In a murine model of CRS, NK cell depletion caused an exacerbation of blood eosinophilia and eosinophilic inflammation in the sinonasal tissue. PGD_2_ and its metabolite, but not PGE_2_ and a panel of cytokines including TGF-β, were increased in CRS patients compared with controls. Effector functions of NK cells were potently suppressed by PGD_2_-dependent, rather than PGE_2_-dependent, pathway in controls and CRS patients. Thus, our results suggest decreased NK cell-mediated eosinophil regulation, possibly through an increased level of PGD_2_, as a previously unrecognized link between PG dysregulation and eosinophilic inflammation in CRS.

Chronic rhinosinusitis (CRS) is a heterogeneous inflammatory upper airway disease characterized by infiltration of inflammatory cells into the sinonasal mucosa. Eosinophilic inflammation is a major pathologic feature of CRS, especially CRS with nasal polyps (CRSwNP)^1^,^2^,^3^. Persistent eosinophilic inflammation is related to prolonged survival of eosinophils as well as their accumulation in tissues^4^,^5^,^6^. In patients with allergic sinusitis, eosinophils accumulate in the superficial lamina propria, where their apoptosis can be detected^6^.

Recently, immune regulatory function of natural killer (NK) cells on other inflammatory cells, particularly eosinophils, is being actively investigated^7^,^8^,^9^,^10^,^11^. NK cells are involved in regulating the activation and apoptosis of inflammatory cells, such as neutrophils and eosinophils^8^,^9^,^10^. Furthermore, NK cells play a role in the recognition and clearance of eosinophils in the airway of asthmatic mice^11^. We previously reported that the effector functions of peripheral blood NK cells, including degranulation and production of interferon (IFN)-γ and tumor necrosis factor (TNF)-α, are decreased in CRS patients. In addition, these reduced functions of NK cells correlate inversely with blood eosinophil counts^12^. Peripheral blood eosinophilia is well known to be related to tissue eosinophilia and recurrence of CRS after surgery^13^,^14^,^15^. These findings suggest that the immune regulatory function of NK cells may play a role in regulating the eosinophilic inflammation in CRS.

Prostaglandin (PG) derived from arachidonic acid is produced in most tissues and organs and has various physiological effects, such as regulation of inflammation. Overexpression of PGD_2_ synthase (PGDS) leads to overproduction of PGD_2_ and promotes eosinophilic, not neutrophilic, lung inflammation in an asthma mouse model^16^. PGDS expression is increased in nasal polyps (NPs) and positively correlates with eosinophilic inflammation^17^. The concentration of PGD_2_ is also elevated in NPs and strongly correlates with the number of mast cells that mainly produce PGD_2_ and play crucial pathogenic roles in CRSwNP^18^. Thus, PGD_2_ may be an important contributing factor to eosinophilic inflammation of CRS. Furthermore, PGD_2_ has been reported to suppress cytotoxicity and IFN-γ and TNF-α production in NK cells^19^. We speculated therefore that the increased PGD_2_ level and decreased NK cell function observed in patients with CRS may be associated with eosinophilic inflammation in the sinonasal tissue and blood eosinophilia. In our present study, we obtained evidence indicating that NK cell dysfunction is potentially linked to PGD_2_ dysregulation and eosinophilic inflammation in CRS.

## Results

### NK cell-mediated eosinophil apoptosis is decreased in CRS patients

**Figure 1:**
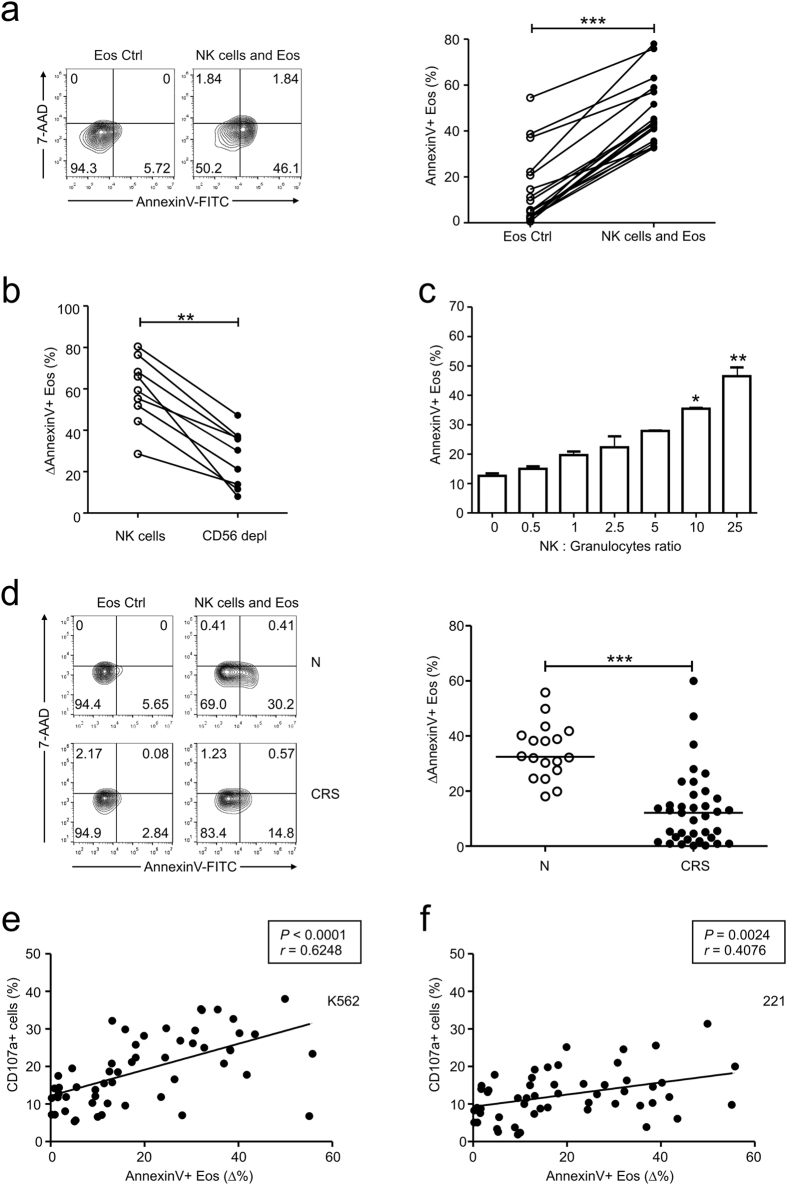
NK cell effector function correlates with eosinophil apoptosis. (**a**–**c**) Peripheral blood granulocytes from the controls were incubated with autologous PBMCs (**a**), autologous CD56-depleted lymphocytes (**b**), or purified NK cells (**c**). (**a**) Representative FACS profiles (*left panel*) and statistical dot plots (*right panel*) showing the percentage of annexin V+ eosinophils. (**b**) Statistical dot plots showing the percent increase in annexin V+ eosinophils after incubation with effector cells relative to that without effector cells (ΔAnnexin V+ Eos). (**c**) Statistical bar charts showing the percentage of annexin V+ eosinophils. (**d**) PBMCs from the controls (N) (n = 18) or patients with CRS (n = 37) were incubated with autologous peripheral blood granulocytes. Representative FACS profiles (*left panel*) and statistical dot plots (*right panel*) showing the relative percent increase in eosinophil apoptosis (ΔAnnexin V+ Eos). (**e,f**) The relative percent increase in eosinophil apoptosis (ΔAnnexin V+ Eos) correlated with the percentages of CD107a+ NK cells after stimulation with K562 (**e**) or 221 (**f**) cells. Horizontal bars indicate the medians. **P < 0.01; ***P < 0.001, Wilcoxon matched-pairs signed-rank test (**a,b**), repeated measure ANOVA with the Friedman’s test (**c**), Mann-Whitney *U* test (**d**), and Spearman correlation test (**e,f**).

We first evaluated eosinophil apoptosis by annexin V and 7-AAD staining after a 4-h incubation of freshly isolated granulocytes with autologous peripheral blood mononuclear cells (PBMCs). Compared with the control group, there was a significant increase in eosinophil apoptosis in granulocytes cultured with PBMCs (Fig. 1a, Supplementary Fig. S1). To determine whether eosinophil apoptosis was primarily mediated by NK cells or a general capacity shared by other lymphocytes in PBMCs, a CD56-depleted lymphocyte population was used in the apoptosis experiments (Supplementary Fig. S2). CD56-depleted lymphocytes exhibited a significant decrease in triggering eosinophil apoptosis, suggesting that the ability to induce eosinophil apoptosis is mostly confined to NK cells (Fig. 1b). In support of this, purified NK cells significantly increased eosinophil apoptosis in the co-culture experiments in a dose-dependent manner (Fig. 1c).

Given the decreased effector functions of NK cells in CRS patients^12^, we hypothesized that NK cell-mediated eosinophil apoptosis may be dysfunctional in CRS patients. NK cells from our study subjects with CRS (Table 1) exhibited a significant decrease in NK cell degranulation after stimulation with K562 target cells (Supplementary Fig. S3a). Peripheral blood eosinophil counts were also significantly higher in CRS patients than in healthy controls (Supplementary Fig. S3b) and correlated inversely with the frequencies of CD107a+ NK cells (Supplementary Fig. S3c), confirming an inverse relationship between NK cell function and blood eosinophil count^12^. Hence, we next compared NK cell-mediated apoptosis of autologous eosinophils between CRS patients and healthy controls. NK cells from CRS patients exhibited a significantly reduced capacity to induce eosinophil apoptosis (Fig. 1d). Interestingly, the apoptosis of eosinophils positively correlated with the frequencies of CD107a+ NK cells after stimulation with both K562 (Fig. 1e) and 221 (Fig. 1f) target cells, suggesting a correlation between NK cell effector function and eosinophil apoptosis.

### Eosinophilic inflammation is associated with decreased NK cell function

**Figure 2:**
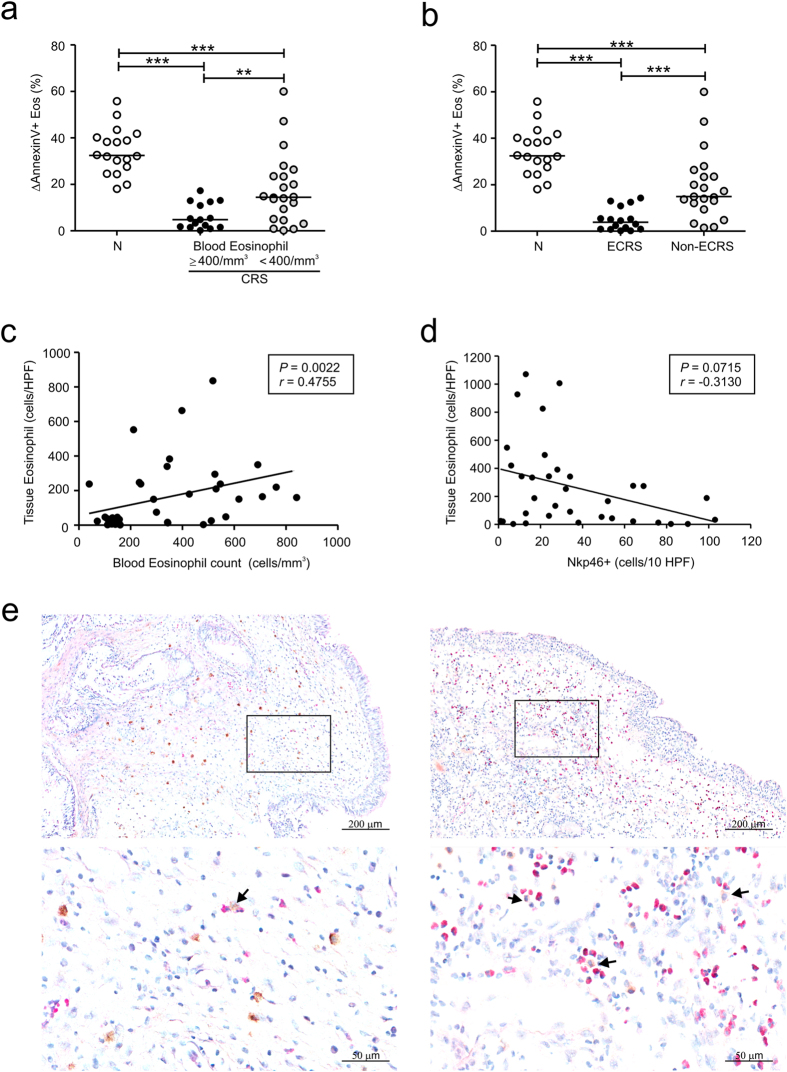
Eosinophilic inflammation is associated with reduced NK cell function in CRS. (**a**,**b**) Patients with CRS were divided into eosinophilic (n = 15) and noneosinophilic (n = 22) groups on the basis of blood eosinophil counts (**a**) or eosinophilic CRS (ECRS) (n = 16) and non-ECRS (n = 21) groups based on tissue eosinophil counts (**b**). PBMCs from each group were incubated with autologous peripheral blood granulocytes. The three groups were compared via statistical dot plots in terms of the relative percent increase in eosinophil apoptosis (ΔAnnexin V+ Eos). (**c**) The peripheral blood eosinophil counts correlated positively with the frequency of eosinophil counts in sinus tissue from CRS patients (n = 39). (**d**) The counts of NKp46^+^ NK cells correlated inversely with the frequency of eosinophil counts in sinus tissue from CRS patients (n = 34). (**e**) Representative immunostaining for NKp46^+^ NK cells and hematoxylin and eosin counterstaining in sinonasal tissue from patients with non-ECRS (*left panel*) and ECRS (*right panel*). Arrows denote NKp46^+^ NK cells in contact with or close to eosinophils. Scale bars represent 200 μm (*top panel*) and 50 μm (*bottom panel*). Horizontal bars denote the medians. ***P < 0.001, Mann-Whitney *U* test (**a,b**) and Spearman correlation test (**c,d**).

Next, we evaluated the relationship between NK cell function and eosinophilic inflammation in peripheral blood and the sinonasal mucosa. To investigate blood eosinophilia, 43 CRS patients were divided on the basis of their blood eosinophil counts into two subgroups, namely, an eosinophilic group (≥400/mm^3^ cutoff point; n = 15) and a noneosinophilic group (<400/mm^3^ cutoff point; n = 28)^20^. Compared with the control group, the eosinophilic group exhibited a marked reduction in NK cell-mediated eosinophil apoptosis (Fig. 2a). This decrease was more pronounced than the decrease in the noneosinophilic group. Next, the association of tissue eosinophilia with NK cell function was assessed by dividing the CRS patients into two study groups based on the tissue eosinophil counts in a visual field per section. Eosinophilic CRS (ECRS) was defined as CRS with eosinophil counts of more than 100 cells/high-powered field (HPF) in sinus tissue^21^,^22^. There were 18 patients in the ECRS group and 25 in the non-ECRS group (Table 2). Compared with the control group, the ECRS group exhibited a severe decrease in NK cell-mediated eosinophil apoptosis, whereas this decrease was less significant in the non-ECRS group (Fig. 2b). Thus, the patients with blood or tissue eosinophilia exhibited the most severe decrease in NK cell-mediated eosinophil apoptosis, suggesting an association between reduced NK cell function and eosinophilic inflammation in CRS.

Consistent with previous studies^22^,^23^,^24^, there was a positive correlation between the numbers of peripheral and tissue eosinophils in CRS patients (Fig. 2c). Moreover, eosinophil counts in the sinonasal mucosa correlated inversely with the numbers of NKp46-positive NK cells in the same tissue sections (Fig. 2d), suggesting a role of NK cells in the regulation of eosinophilic inflammation in sinonasal tissue as well as in peripheral blood. Supporting this, NK cells in contact with or in close proximity to eosinophils were detected in the sinonasal mucosa of CRS patients (Fig. 2e).

### NK cells regulate eosinophilic inflammation in a murine model of CRS

**Figure 3:**
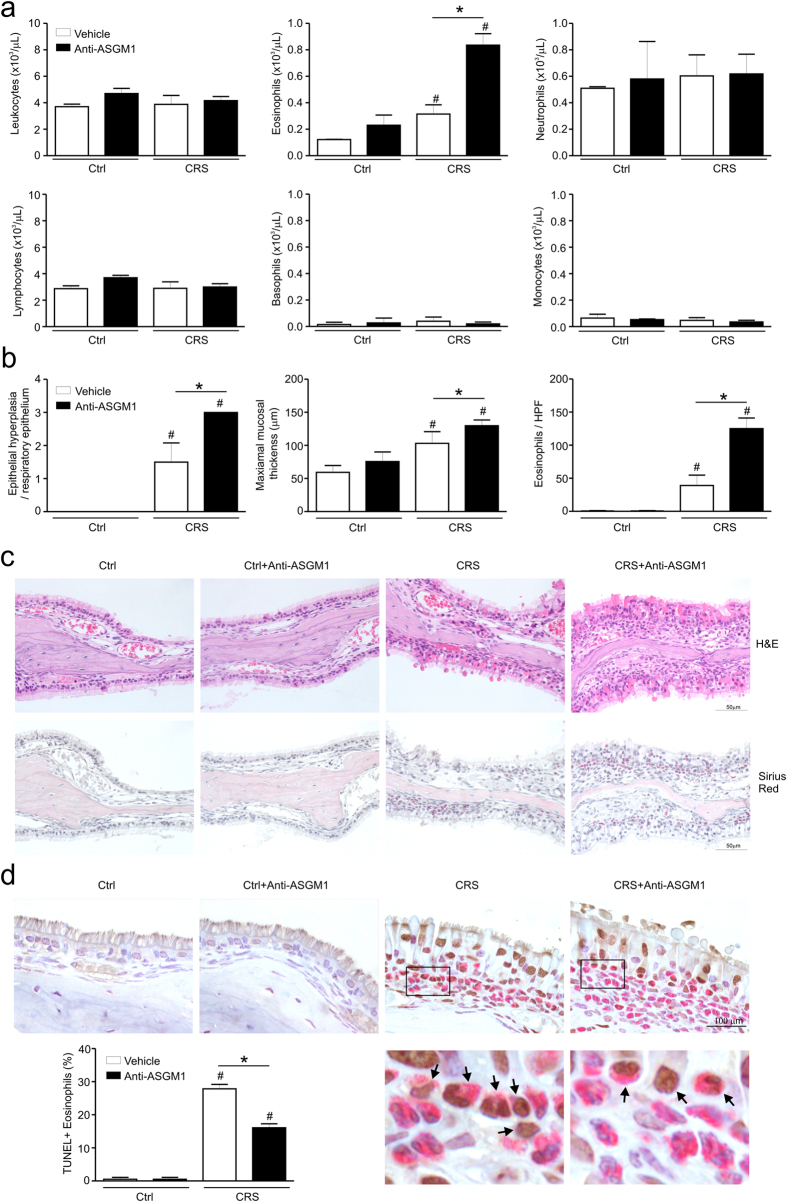
NK cells regulate eosinophilic inflammation in a murine model of CRS. (**a**) Counts of leukocytes, eosinophils, neutrophils, lymphocyte, basophils, and monocytes in blood from each group of mice (n = 4). (**b**) Scores of epithelial hyperplasia (*left*), maximal mucosal thickness (*middle*) in hematoxylin and eosin-stained tissue sections, and eosinophil counts of the lamina propria (*right*) in Sirius red-stained tissue sections. (**c**) Representative photographs of hematoxylin and eosin (*upper*, original magnification x400) and Sirius red (*lower*, original magnification x400)-stained sections. (**d**) Representative photographs of TUNEL- and Sirius red-stained sections and quantitative analysis of TUNEL-positive eosinophils. The black box indicates the magnified area. Arrows denote TUNEL-positive eosinophils. Scale bars represent 100 μm. Statistical bar chart shows the percentage of TUNEL-positive cells relative to total eosinophils. Data are expressed as means ± SDs. *P < 0.05; ^#^*P* < 0.05, Mann-Whitney *U* test (**a**,**b**,**d**).

To directly probe the role of NK cells in the eosinophilic inflammation of CRS, we used a CRS mouse model^25^ and investigated the severity of blood and tissue eosinophilia by NK cell depletion with anti-asialo ganglio-N-tetraosylceramide 1 (ASGM1) treatment. We found that the number of CD3ε-NKp46^+^ NK cells was lower in CRS mice than in control mice (3.19 ± 0.13% vs. 3.97 ± 0.07%, Supplementary Fig. S4a,b). The CD107a expression of the CD3ε-NKp46^+^ NK cells was also decreased in CRS mice compared with control mice (23.15 ± 0.87% vs. 30.67 ± 1.45%, Supplementary Fig. S4c,d). After anti-ASGM1 treatment, the number of CD3ε-NKp46^+^ NK cells in both control and CRS mice was significantly decreased (Supplementary Fig. S5). The blood eosinophils in control mice were not changed by anti-ASGM1 treatment, whereas the blood eosinophils in CRS mice were significantly increased by NK cell depletion with anti-ASGM1 treatment (Fig. 3a). Histological analyses revealed that epithelial hyperplasia and maximal mucosal thickness were more pronounced in CRS mice treated with anti-ASGM1 compared with untreated CRS mice (*P* < 0.05, Fig. 3b,c). The number of eosinophils in the sinonasal mucosa was also significantly higher in CRS mice treated with anti-ASGM1 compared with untreated CRS mice (*P* < 0.05, Fig. 3b,c). Thus, these results indicated an inhibitory effect of NK cells on blood and tissue eosinophilia in a murine model of CRS. Of note, the percentage of apoptotic eosinophils relative to total eosinophils was significantly lower in CRS mice after anti-ASGM1 treatment compared with untreated CRS mice (*P* < 0.05, Fig. 3d), suggesting an association of NK cell depletion with eosinophil accumulation through an effect on eosinophil apoptosis.

### PGD_2_ may contribute to decreased NK cell function in CRS

**Figure 4:**
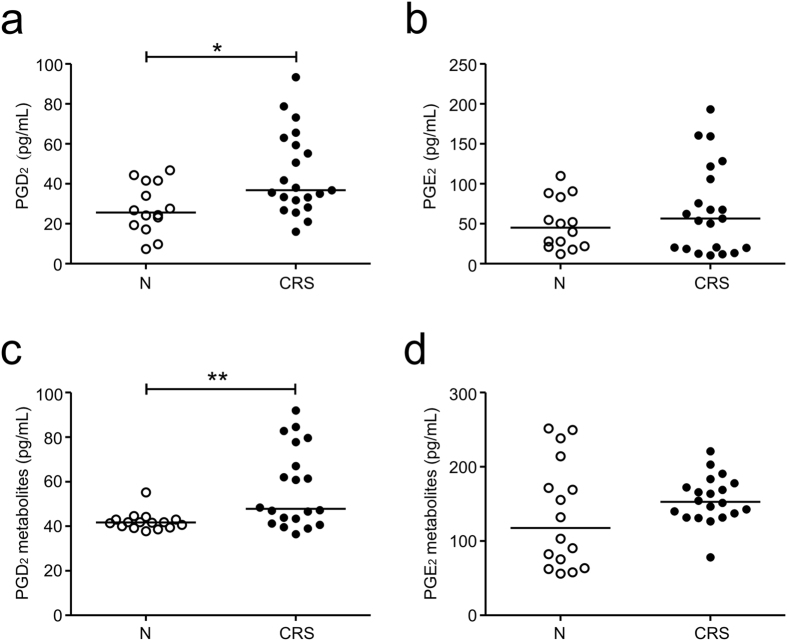
Patients with CRS have altered levels of PGD_2_. (**a**,**b**) PGD_2_ (**a**) and PGE_2_ (**b**) levels in the serum from the controls (N) (n = 14) or patients with CRS (n = 21) were measured by using LC-MS/MS. (**c**,**d**) Levels of PGD_2_ metabolite (**c**) and PGE_2_ metabolite (**d**) in the serum from the controls (N) (n = 16) or patients with CRS (n = 20) were measured by using an enzyme immunoassay. Horizontal bars denote the medians. *P < 0.05; **P < 0.01, Mann-Whitney *U* test.

CRS is a persistent inflammatory disease associated with an elevation of diverse inflammatory cytokines and molecules^26^. To probe soluble mediator(s) that contribute to reduced NK cell function in CRS, we assessed the levels of various cytokines in the serum of the CRS patients and control subjects using a multiplex cytokine assay. The two groups did not differ significantly in terms of the production of inflammatory cytokines, namely, IFN-γ, IL-2, IL-4, IL-5, IL-10, IL-12, IL-13, IL-15, IL-17, and TGF-β (Supplementary Fig. S6). Because several studies have shown that PGs contribute to the suppression of NK cell function^19^,^27^, we determined the levels of serum PGs using LC-MS/MS. Compared with the control group, the CRS group had significantly higher levels of PGD_2_ but not PGE_2_ (Fig. 4a,b). To confirm this finding, we measured the levels of PG metabolites by using an enzyme immunoassay. The levels of PGD_2_ metabolite (11β-PGF_2α_) but not PGE_2_ metabolites (13,14-dihydro-15-keto metabolites) were also significantly elevated in the CRS group (Fig. 4c,d). In comparison, the levels of PGE_2_ and its metabolites were slightly elevated but not significantly different in the CRS group. Thus, PGD_2_ may be a potential candidate mediator implicated in NK cell dysfunction in CRS, compatible with an elevated expression of PGDS and production of PGD_2_ in CRS^17^,^18^.

### PGD_2_ attenuates NK cell effector functions

**Figure 5:**
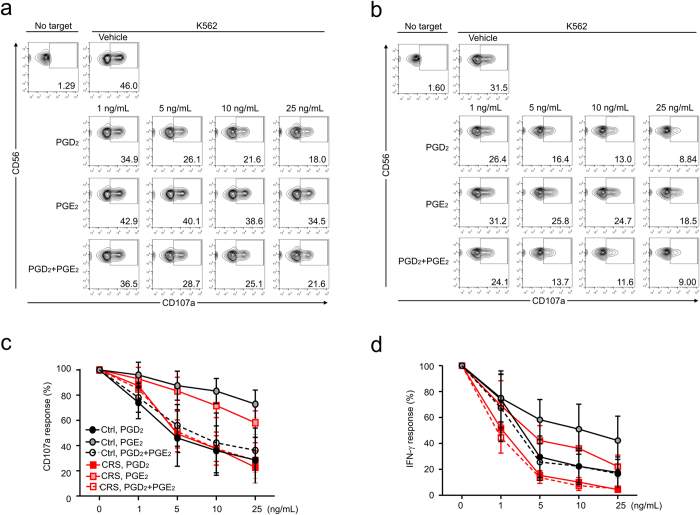
PGs attenuate the effector functions of NK cells. PBMCs were pretreated with PGD_2_, PGE_2_, or a combination of PGD_2_ and PGE_2_ and incubated with K562 cells. (**a**,**b**) Representative FACS profiles showing the effect of PGs on the expression of CD107a on NK cells from the controls (**a**) or patients with CRS (**b**). (**c**) Line graphs showing the percent response of CD107a+ NK cells from the controls (black) or CRS patients (red) with different concentrations of PGs relative to that with vehicle only. (**d**) Line graphs showing the percent response of intracellular IFN-γ+ NK cells from the controls (black) or CRS patients (red) at varying PG concentrations relative to that with vehicle only. Data are expressed as means ± SDs.

To evaluate whether PGs affect NK cell effector functions, we first assessed the effect of PGs on NK cell degranulation upon stimulation with K562 cells. PBMCs from healthy controls were incubated with target cells for 2 h after pretreatment with PGD_2_, PGE_2_, or both for 1 h. PGD_2_ rather than PGE_2_ potently inhibited the degranulation of NK cells against K562 target cells in a dose-dependent manner (Fig. 5a,b). The combination of PGD_2_ and PGE_2_ did not have an additive effect on the reduction of NK cell degranulation. The observation made in NK cells from healthy controls was also applicable to NK cells from CRS patients; a similar and marked attenuation of NK cell degranulation in response to PGD_2_ or its combination with PGE_2_ but a slightly increased sensitivity to PGE_2_ in the attenuation of NK cell degranulation (Fig. 5c,d). Next, we assessed the effect of PGs on the NK cell production of the inflammatory cytokine IFN-γ. The intracellular expression of IFN-γ was measured in NK cells from healthy controls (Fig. 5e) and CRS patients (Fig. 5f) after stimulation with K562 cells. As observed with NK cell degranulation, NK cell production of IFN-γ was markedly and dose-dependently reduced by PGD_2_ and less potently by PGE_2_ (Fig. 5e,f). These data suggest that reduced NK cell function in CRS is not associated with differential sensitivity of CRS NK cells to PGD_2_ but likely with PGD_2_ dysregulation, although we cannot exclude the possible contribution of PGE_2_.

To gain an insight into the mechanism of action of PGs on NK cells, we first assessed the expression of NK cell-activating receptors, namely, CD16, 2B4, DNAM-1, NKp46, NKp44, NKp30, NKG2D, and NKG2C. The expression of activating receptors on NK cells was nearly unaffected after treatment with PGD_2_ (Supplementary Fig. S7). The expression of 2B4, NKp44, and NKG2D was slightly decreased by PGE_2_. These results suggest that PG-mediated NK cell dysfunction, in particular that mediated by PGD_2_, is not primarily associated with defective expression of activating receptors. In comparison, the expression levels of perforin and granzyme B, critical mediators of target cell killing following NK cell degranulation, were significantly decreased by PGD_2_ (Supplementary Fig. S8). Furthermore, given an increased cAMP level upon exposure to PGs and its association with NK cell dysfunction^19^,^28^, the involvement of the cAMP pathway was investigated using Rp-8Br-cAMP, an inhibitory cAMP analog^29^. Rp-8Br-cAMP significantly prevented the PG-induced NK cell dysfunction against K562 target cells in terms of NK cell degranulation (Supplementary Fig. S9a,b) and IFN-γ expression (Supplementary Fig. S9c,d). Moreover, Rp-8Br-cAMP also reversed the PG-induced NK cell dysfunction with dose dependence (Supplementary Fig. S10). Taken together, our data suggest that functional deficiencies of NK cells in response to PGs, particularly PGD_2_, are likely due to reduced levels of cytotoxic mediators and a signaling defect involving the cAMP pathway downstream of activating receptors.

### PGD_2_ attenuates NK cell-mediated eosinophil apoptosis

**Figure 6:**
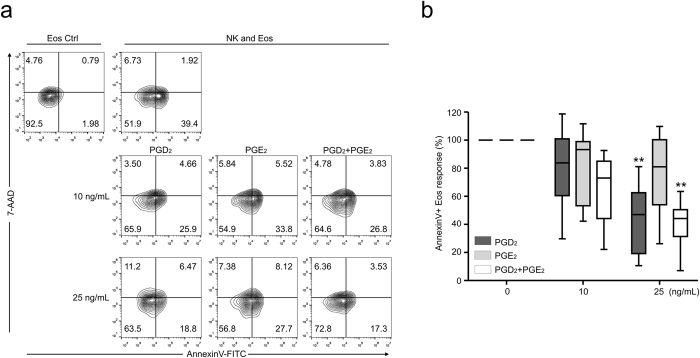
PGs attenuate NK cell-mediated eosinophil apoptosis. PBMCs were pretreated with PGD_2_, PGE_2_, or combination of PGD_2_ and PGE_2_ and then incubated with autologous granulocytes. (**a**) Representative FACS profiles showing the effect of PGs on the percentage of annexin V+ eosinophils. (**b**) Statistical line graphs showing the percent response of annexin V+ eosinophils to different concentrations of PGs relative to that with vehicle only. Box and whisker plots show the median and the minimum to maximum percentiles. *P < 0.01; **P < 0.001 by repeated measure ANOVA with the Friedman’s test.

Finally, we evaluated whether PGs could modulate NK cell-mediated eosinophil apoptosis. Eosinophil apoptosis was assessed after 4-h incubation of granulocytes with autologous PBMCs pretreated with PGD_2_, PGE_2_, or a combination of PGD_2_ and PGE_2_ for 1 h. NK cell-mediated eosinophil apoptosis was significantly blocked in the presence of PGD_2_ but not PGE_2_ with dose dependence (Fig. 6a,b). Although we observed some additive effect of PGE_2_ on blocking NK cell-mediated eosinophil apoptosis, the statistical significance was not consistent. PGD_2_, PGE_2_, or a combination of PGD_2_ and PGE_2_ per se did not affect eosinophil apoptosis for 4 h, thus ruling out a toxic effect of PGs on eosinophils during the time frame of the assay (Supplementary Fig. S11). PGD_2_ acts through two different receptors, D prostanoid receptor 1 (DP1, also called DP) and DP2 [also termed chemoattractant receptor-like molecule on the Th2 cell (CRTH2)]. NK cell-mediated eosinophil apoptosis was significantly blocked by an agonist of DP1 (BW245C) but not an agonist of DP2 (DKPGD_2_) (Supplementary Fig. S12), an observation compatible with the inhibition of NK cell functions by DP1 rather than DP2 agonist^19^. Taken together, our results suggest that PGD_2_, whose level is frequently elevated in CRS, rather than PGE_2_, may be an important contributing factor in aggravating eosinophilic inflammation in CRS by reducing NK cell function via DP1 and NK cell-mediated eosinophil regulation.

## Discussion

NK cells are cytotoxic innate lymphoid cells that are critical for the innate immune system and play an important role in primary defense against viral infection and cancer cells. In addition, NK cells have immunomodulatory functions on other immune cells, such as dendritic cells, T cells, B cells, monocytes, and neutrophils, through cell-cell contacts and production of various chemokines and cytokines^10^,^30^. Our current study findings demonstrate, for the first time, that NK cell-induced eosinophil apoptosis is decreased in patients with CRS and that a depletion of NK cells in a CRS mouse model aggravates eosinophilic sinonasal inflammation and blood eosinophilia via an effect on eosinophil apoptosis. In addition, the findings of an increased level of PGs, particularly PGD_2_, in patients with CRS and a potent suppression of NK cell function by PGD_2_ may provide a potential link among PG dysregulation, NK cell dysfunction, and eosinophilic inflammation.

Eosinophilic inflammation in CRS is frequently related to more severe CRS and recurrence of NPs, and it has been considered to be a major therapeutic target^31^,^32^. In support of this, the strategy that promotes eosinophil apoptosis has been pursued as a novel therapeutic modality for the resolution of eosinophilic inflammation in asthma^33^. In general, eosinophilic inflammation in CRS has been primarily understood in the context of a disorder of the adaptive immune system, including effector T cells and their associated cytokines^31^,^34^. In this respect, our results of eosinophil regulation by NK cells supports an innate immunoregulatory role for NK cells and suggest a decreased function of NK cells observed in CRS as a previously unrecognized mechanism that promotes eosinophilic inflammation in CRS.

The crosstalk between NK cells and eosinophils has been demonstrated previously. NK cells from patients with allergic rhinitis showed chemotactic activity against eosinophils^35^. In severe asthmatic patients, the total number of peripheral blood NK cells and capacity of NK cell-induced eosinophil apoptosis were reduced, although there was a positive correlation between the percentage of peripheral blood eosinophils and that of NK cells or the extent of NK cell activation^9^. Co-culture of NK cells and autologous eosinophils increased mitochondrial reactive oxygen species in eosinophils and induced eosinophil apoptosis in a contact-dependent manner regardless of the allergic status of the subjects^10^. In CRS patients, decreased cytotoxicity and IFN-γ production of NK cells correlated inversely with blood eosinophil counts^12^. In our present study, when eosinophils were incubated with autologous NK cells, peripheral blood NK cell-induced eosinophil apoptosis was significantly reduced in CRS patients compared with healthy controls. In particular, this decrease was most severe in the CRS patients with blood or sinonasal eosinophilia. The degranulation capacity of NK cells also positively correlated with eosinophil apoptosis in peripheral blood from CRS patients. These results suggest that decrease in NK cell-mediated eosinophil regulation might be associated with prolonged survival of eosinophils and their accumulation in CRS patients. In addition, these results are in accordance with the results of a previous study showing that eosinophil apoptosis is directly induced by NK cells^10^.

Because NK cells constitute ~10% to 15% of peripheral blood lymphocytes and are not abundant in sinus tissue^36^, NK cells in the tissue from CRS patients were counted in 10 HPFs, particularly due to the sparse population of NK cells in ECRS samples. The potential interaction between NK cells and eosinophils was detected in the sinonasal mucosa of CRS patients. Furthermore, NKp46^+^ NK cells in the sinus tissue correlated negatively with tissue eosinophils in terms of their number and distribution. These findings support the notion that NK cells may have the capacity to regulate the accumulation of eosinophils in sinus tissue as well as blood eosinophilia. El-Shazly *et al*.^37^ reported that NK cell infiltration was significantly increased in patients with allergic CRS (patients with a long lasting and poorly controlled allergic rhinitis) without asthma compared with controls, non-allergic CRS, or allergic CRS with asthma patients. Although the two sets of data may not be directly comparable, their results suggest a functional interaction between NK cells and eosinophils in allergic rhinitis patients.

To confirm the crosstalk between NK cells and eosinophils in CRS, we depleted NK cells by anti-ASGM1 treatment in an eosinophilic CRS mouse model created by a combination of *Aspergillus* protease (AP) and ovalbumin (OVA) and evaluated the change in blood eosinophilia and tissue eosinophilic inflammation. Protease from fungi has been implicated in the pathogenic mechanism for allergic airway diseases such as NPs by protease-activated receptor (PAR)-driven proinflammatory cytokine production and eosinophil recruitment^38^,^39^. Thus, our CRS mouse model based on the combination of fungal protease and OVA better represents human eosinophilic CRS in terms of an actual triggering factor-induced model and by sharing many histological and immunological features with eosinophilic CRS^2^,^25^. In fact, this model shows chronic eosinophilic sinonasal inflammation, including epithelial hyperplasia, subepithelial eosinophil infiltration, goblet cell hyperplasia, and Th2 polarization^25^. In our current study, eosinophilic CRS mice showed decreases in the number and degranulation of NK cells compared with control mice. Furthermore, NK cell depletion in the eosinophilic CRS mice led to the aggravation of eosinophilic inflammation in the blood as well as sinus tissue and the decrease of eosinophil apoptosis in the sinus tissue. Recent study demonstrated that NK cells play an important role in the timely resolution of allergic airway inflammation^40^. Supporting this notion, NK cell depletion after CRS development led to persistent eosinophilic inflammation in the blood and sinus tissue (Supplementary Fig. S13). In comparison, CRS mice in the absence of NK cell depletion showed a relative decrease in eosinophilic inflammation both in the sinus tissue and, in particular, blood. Thus, we speculate that NK cells contribute to the resolution of eosinophilic inflammation in CRS although the exact mechanism underlying such proresolving role of NK cells remains to be determined. Collectively, these results suggest that the reduction of NK cell function can promote eosinophilic inflammation in the blood and sinonasal tissue in a murine CRS model, appropriately reflecting the association between NK cell dysfunction and eosinophilic inflammation in CRS patients.

It has been shown that PGD_2_ and PGE_2_ can suppress NK cell cytolytic function and IFN-γ production via a cAMP-dependent pathway^19^,^27^. Given comparable serum levels of a panel of inflammatory cytokines between the controls and CRS patients, we assumed that PGs would be involved in the reduction of NK cell-induced eosinophil apoptosis in CRS patients. We observed that the levels of PGD_2_ and its metabolite, but not PGE_2_, were elevated in serum from the CRS patients and that NK cell degranulation and IFN-γ production were significantly attenuated by treatment with PGD_2_. Furthermore, this PGD_2_-induced suppression of NK cell functions was also dependent on the cAMP pathway in CRS patients. However, given the decreased levels of cytotoxic mediators (e.g., perforin and granzyme B) in NK cells exposed to PGD_2_, the involvement of other regulatory mechanism(s) cannot be excluded.

PGD_2_ functions through two receptors, DP1 and DP2 and is important for Th2-mediated inflammatory disease via the recruitment of Th2 cells, eosinophils, and basophils. In an allergic asthma mouse model, PGD_2_ and PGDS have been implicated in triggering an allergic response, such as eosinophilia, airway hyperreactivity, mucus production, and increased abundance of Th2 cytokines^16^,^17^. In CRSwNP patients, the levels of PGDS mRNA expression in inflamed sinus mucosa were elevated, independently of atopic status. The increased PGDS may induce increased production of PGD_2_, resulting in recruitment and activation of Th2 cells and eosinophils via a DP2 that promotes eosinophilic inflammation^41^,^42^. Thus, increased PGD_2_ levels may be involved in eosinophilic inflammation regardless of atopic status in CRS patients. Allergic asthma patients, whose basal levels of the plasma PGD_2_ metabolite were similar to those of non-allergic controls, exhibited a significant increase in plasma PGD_2_ metabolite levels upon allergen challenge^43^.

In our present study, PGD_2_ and its metabolite were increased in the serum of active CRS patients and NK cell effector functions were potently suppressed upon exposure to PGD_2_ and an agonist of DP1 (BW245C). These results are in accordance with the results of a previous study showing that PGD_2_ suppresses NK cell function via DP1 signaling^19^. In this respect, PGD_2_ may play an important role in the modulation of eosinophilic inflammation through a suppression of NK cell effector function, beyond its direct proinflammatory and chemotactic effects on eosinophils. Recent study demonstrated that PGD_2_ also suppresses chemotaxis of human NK cells^19^, which could be a possible explanation for the inverse correlation between eosinophil counts and NKp46-positive NK cell counts in the sinonasal mucosa. Thus, we speculate that suppressing effect of PGD_2_ on NK cell effector function and migration to the site of inflammation may together contribute to promoting eosinophilic inflammation in CRS.

In comparison, the levels of PGE_2_ and its metabolite were comparable between CRS patients and control subjects, and PGE_2_-induced suppression of NK cell function and prevention of NK cell-mediated eosinophil apoptosis were insignificant. Thus, PGE_2_ may not play a primary role in NK cell dysfunction in CRS patients, compatible with the inverse correlation between PGE_2_ levels and eosinophilic inflammation in CRS patients^17^,^44^.

In summary, we here show a previously unappreciated aspect of a proinflammatory effect of PGD_2_ on the pathophysiology of CRS in the context of NK cell dysfunction. Reduced eosinophil apoptosis and consequent eosinophilic inflammation in CRS patients may be attributed to a PGD_2_ dysregulation and a consequent NK cell dysfunction. In support of an immune regulatory function of NK cells on other inflammatory cells, our results support the notion that NK cells play a significant role in eosinophilic inflammation in CRS pathogenesis. Our findings also suggest that suppression of PGD_2_ and recovery of NK cell function may be potential therapeutic targets in eosinophilic inflammation of CRS.

## Methods

### Study subjects and sample collection

Forty-three adult patients who underwent endoscopic sinus surgery for CRS were recruited from the Department of Otolaryngology, Asan Medical Center, between May 2013 and October 2014. All patients with CRS fulfilled the established diagnostic criteria for CRS as defined by the American Academy of Otolaryngology–Head and Neck Surgery Chronic Rhinosinusitis Task Force^45^. Patients with fungal sinusitis, classic allergic fungal sinusitis, aspirin-exacerbated respiratory disease, immunodeficiency, pregnancy, coagulation disorder, or cystic fibrosis were excluded from the study. No patients had received intranasal and/or oral corticosteroids for at least 4 weeks prior to the endoscopic sinus surgery. Peripheral blood samples were collected from patients before surgery in the operating room and used to test NK cell functions and to measure the eosinophil population and apoptosis and concentrations of PGs and cytokines (IFN- γ, IL-2, IL-4, IL-5, IL-10, IL-12p70, IL-13, IL-15, IL-17, and TGF-β). Uncinate tissues from 6 patients with CRS without NPs and NPs from 37 patients with CRSwNP were obtained during endoscopic sinus surgery and used to quantify eosinophils and NK cells. The control subjects were 18 healthy volunteers at least 18 years of age who agreed to donate their blood for research purposes. They did not have any symptoms and objective evidence of sinonasal disease. Clinical and demographic findings of patients and control subjects are summarized in Tables 1 and 2. This study was approved by the Institutional Review Board of Asan Medical Center and all participants provided written informed consent.

### Ethics statement

Human study in this work was approved by the Institutional Review Board (IRB) of Asan Medical Center and carried out in accordance with the relevant guidelines set forth by IRB, and all participants provided written informed consent. All animal experiments were approved by Institutional Animal Care and Use Committee (IACUC) of Asan Institute for Life Sciences and done in accordance with the approved guidelines set forth by IACUC.

### Quantification of eosinophils and NK cells in tissue

The infiltration of eosinophils and NK cells in the sinus tissue was evaluated in a blind manner by a pathologist in the Department of Pathology, Asan Medical Center. The number of eosinophils in the lamina propria per HPF was counted in hematoxylin and eosin-stained sections using light microscopy (x400 magnification). Three HPFs containing the greatest degree of cellular infiltration were assessed for each biopsy, and then the scores were averaged for each patient. The population of NK cells in the lamina propria was determined by immunohistochemistry for NKp46, a NK cell receptor. Counterstaining was performed with eosin to assess the interaction between NK cells and eosinophils in the sinus tissues. Formalin-fixed, paraffin-embedded tissue sections were immunohistochemically stained with anti-NKp46 antibody using a BenchMark XT automated immunostaining device (Ventana Medical Systems, Tucson, AZ) with an ultraView Universal DAB Detection Kit (Ventana Medical Systems) according to the manufacturer’s instructions. Briefly, 4-μm-thick sections, obtained with a microtome, were transferred onto silanized charged slides and allowed to dry for 10 min at room temperature, followed by 20 min in an incubator at 65 °C. Sections were treated with alkaline protease (Protease 1; Ventana Medical Systems) for 1 min to uncover antigenic sites and incubated for 16 min with monoclonal anti-NKp46 (CD335) (1:25; BD Pharmingen, San Jose, CA) in the autoimmunostainer, before being dipped in eosin for 1 min. The number of NK cells was counted per 10 HPFs containing the greatest degree of eosinophil infiltration.

### Reagents and antibodies

PGD_2_, PGE_2_, BW245C (DP1-specific agonist) and DKPGD2 (DP2-specific agonist) were purchased from Cayman Chemical (Ann Arbor, MI) and Rp-8Br-cAMP was purchased from Calbiochem (Darmstadt, Germany). The following monoclonal antibodies were used: anti-human CD3-PerCP (SK7), anti-human CD3-APC (UCHT1), anti-human CD19-APC (HIB19), anti-human CD16-PE (3G8), anti-human CD56-PE (NCAM16.2), anti-human CD107a-FITC (H4A3), anti-human IFN-γ-FITC (25723.11), anti-human DNAM-1-PE (DX11), anti-human NKp46-PE (9E2), anti-human NKp44-PE (p44-8), anti-mouse CD3ε-PerCP (145-2C11), anti-mouse NKp46-PE (29A1.4), anti-mouse CD107a-FITC (1D4B), anti-mouse IFN-γ-FITC (XMG1.3), and anti-mouse CD16/CD32 (mouse Fc Block; 2.4G2) from BD Biosciences (San Jose, CA); anti-human 2B4-PE (C1.7) and anti-human NKp30-PE (Z25) from Beckman Coulter; anti-human NKG2C-PE (134591) and anti-human NKG2D-PE (149810) from R&D Systems; anti-human perforin-Alexa 647 (dG9) and anti-human granzyme B-Alexa 647 (GB11) from BioLegend. Annexin V-FITC and 7-AAD were from BD Biosciences.

### Cell isolation and culture

Granulocytes were prepared from freshly collected blood samples by a one-step double-density centrifugation method (Lympholyte H and Lympholyte Poly; Cedarlane Laboratories Ltd., Ontario, Canada)^46^. PBMCs were isolated from whole blood by density gradient centrifugation (LSM lymphocyte separation medium; MP Biomedicals). Granulocytes and PBMCs were cultured in RPMI-1640 medium supplemented with 10% fetal bovine serum (FBS), 2 mM L-glutamine, 100 U/mL penicillin, and 100 μg/mL streptomycin. CD56-depleted lymphocytes were obtained from PBMCs by negative selection using a human CD56 positive selection kit (EasySep; Stemcell Technologies). CD56-depleted lymphocytes were assessed by flow cytometric analysis of cells stained with antibodies against CD3 and CD56. The NK cell content of depleted lymphocytes was <1% (Supplementary Fig. S2). In some experiments, human NK cells were purified from PBMCs using an NK cell isolation kit (EasySep, Stemcell Technologies)^47^. The purity of isolated NK cells was assessed by flow cytometric analysis of cells stained against CD3 and CD56. The purity of isolated NK cells was >96%. K562 (ATCC CCL-243) and 721.221 (a gift of J. Gumperz and P. Parham; hereafter referred to as 221) cells were cultured in Iscove’s modified Dulbecco’s medium supplemented with 10% FBS and 2 mM L-glutamine.

### Co-culture experiments

Autologous human granulocytes freshly prepared from peripheral whole blood were incubated at 1 × 10^6^ cells/mL in the absence or presence of PBMCs (1:12.5 ratio of granulocytes vs. PBMCs) for 4 h at 37 °C. Thereafter, the cells were incubated with FcγR-binding inhibitor (eBioscience) for 30 min at 4 °C and stained for surface markers with anti–CD16-PE, anti–CD3-APC, and anti–CD19-APC antibodies for 30 min in the dark at 4 °C. To determine the percentage of apoptotic cells, the cell pellets were resuspended in binding buffer (BD Bioscience) and stained with annexin V and 7-AAD (BD Bioscience) for 15 min in the dark at 25 °C. The cells were then analyzed by flow cytometry. Eosinophils were gated on CD3-CD16-CD19- cells from the granulocyte population on the basis of the forward scatter (FSC) and side scatter (SSC) characteristics (Supplementary Fig. S1).

### Sample preparation for PG measurement

PGD_2_ and PGE_2_ were extracted from 500–1000 μL human serum using solid phase extraction (SPE). A 60-mg Oasis HLB (Waters) SPE cartridge was washed and preconditioned with ethyl acetate, methanol, and 0.1% acetic acid: 5% methanol in H_2_O, sequentially. Ten microliters of 0.2 mg/mL of EDTA and butylated hydroxytoluene in methanol: H_2_O (50:50) were added to the sorbent bed of a SPE column. PGD_2_-d_4_ and PGE_2_-d_4_ were also added as internal standards into samples. Sample solutions were added into the SPE column. The column was washed with 2 column volumes of 0.1% acetic acid: 5% methanol in H_2_O and then dried using a vacuum. Finally, PGD_2_ and PGE_2_ were eluted with 0.5 mL methanol followed by 1.5 mL ethyl acetate. Sample solutions were dried using a vacuum centrifuge, then stored at −20 °C until analysis. The dried matter was reconstituted with 40 μL of 50% acetonitrile prior to LC-MS/MS analysis.

### LC-MS/MS analysis for PG measurement

A LC-MS/MS system equipped with a 1290 HPLC (Agilent), Qtrap 5500 (ABSciex), and reverse phase column (Pursuit 5 200 × 2.0 mm) was used. The separation gradient for PGD_2_ and PGE_2_ analysis used mobile phase A (0.1% acetic acid in H_2_O) and mobile phase B (0.1% acetic acid in acetonitrile/methanol (84/16, v/v) and proceeded at 300 μL/min and 25 °C. The separation gradient was as follows: 40% to 60% of B for 5 min, 60% to 100% of B for 0.1 min, hold at 100% of B for 4.9 min, 100% to 40% of B for 0.1 min, and then hold at 40% of A for 2.9 min. The multiple reaction monitoring mode was used in negative ion mode, and the extracted ion chromatogram corresponding to the specific transition of each analyte was used for quantification (Q1/Q3 = 351/271 and 351/315, retention time [RT] = 4.34 min for PGE_2_; Q1/Q3= 355/275 and 355/319, RT = 4.34 min for PGE_2_-d_4_; Q1/Q3 = 351/271 and 351/315, RT = 4.66 min for PGD_2_; Q1/Q3 = 355/275 and 355/319, RT = 4.66 min for PGD_2_-d_4_;). The calibration range for each analyte was 0.1–1000 nM (r^2^ ≥ 0.99).

### PG treatment of NK cells

To analyze the effect of PG on the function of NK cells, PBMCs were selectively exposed (1 h, 37 °C) to PGD_2_, PGE_2_, or a combination of PGD_2_ and PGE_2_ (10 or 25 ng/mL) before co-incubation with target cells or eosinophils. To assess the involvement of the cAMP pathway in PG-mediated modulation of NK cell function, Rp-8Br-cAMP, a cAMP analog inhibitor, was added to the culture of PBMCs in combination with PGD_2_ (25 ng/mL) and PGE_2_ (25 ng/mL) before co-incubation with target cells. To analyze the effect of PGD_2_ receptor agonists on the function of NK cells, PBMCs were selectively exposed to PGD_2_ (25 ng/mL), BW245C (100 nM), or DKPGD_2_ (100 nM) before co-incubation with eosinophils.

### Assay of NK cell degranulation

NK cell degranulation was determined by the cell surface expression of CD107a, as described^12^. Briefly, PBMCs (2 × 10^5^ cells) were mixed with an equal number of K562 cells and incubated for 2 h at 37 °C. The cell pellets were resuspended in FACS buffer (phosphate-buffered saline [PBS] with 2% FBS) and stained with anti–CD3-PerCP, anti–CD56-PE, and anti–CD107a-FITC antibodies for 30 min in the dark at 4 °C. Lymphocytes were gated on FSC and SSC characteristics, and the CD107a expression on CD3-CD56+ NK cells was analyzed by flow cytometry (BD Bioscience) and FlowJo software (Tree Star).

### Intracellular cytokine staining of NK cells

PBMCs (2 × 10^5^ cells) were stimulated with an equal number of K562 cells for 1 h at 37 °C. Thereafter, brefeldin A (GolgiPlug; BD Bioscience) was added, followed by an additional 5 h of incubation for a total of 6 h. The cells were then stained for surface markers with anti–CD3-PerCP and anti–CD56-PE antibodies for 30 min in the dark at 4 °C. The PBMCs were then washed twice with FACS buffer and incubated in BD Cytofix/Cytoperm solution (BD Bioscience) for 20 min in the dark at 4 °C. Before and after intracellular staining with anti–IFN-γ-FITC for 30 min in the dark at 4 °C, the cells were washed twice with BD Perm/Wash buffer (BD Bioscience). The cells were then analyzed by flow cytometry gated on CD3-CD56+ NK cells.

### Perforin and granzyme B staining of NK cells

PBMCs (2 × 10^5^ cells) were incubated with different concentrations of PGD_2_ (10 or 25 ng/mL) for 5 h at 37 °C. The cells were then stained for surface markers with anti–CD3-PerCP and anti–CD56-PE antibodies. After washing the PBMCs twice with FACS buffer, they were incubated in BD Cytofix/Cytoperm solution. Before and after intracellular staining with anti–perforin-Alexa647 and anti-granzyme B-Alexa 647, the cells were washed twice with BD Perm/Wash buffer and then analyzed by flow cytometry gated on CD3-CD56+ NK cells.

### Murine CRS model

BALB/c mice (6 to 7 weeks old) were purchased from Orient Bio Inc. (Sungnam, Korea). Eosinophilic CRS was induced by intranasal challenge with a mixture of 0.7 U protease from *Aspergillus oryzae* (Sigma-Aldrich, St. Louis, MO) and OVA (Worthington, Lakewood, NJ) diluted in sterile PBS to a total volume of 20 μl 3 times a week for 5 weeks, as previously described with slight modifications^25^. Control mice were challenged intranasally with PBS.

To deplete NK cells, mice were given an intraperitoneal (i.p.) injection of 10 μl rabbit anti-asialo ganglio-N-tetraosylceramide (ASGM1) (Cedarlane Laboratories Ltd.). Control mice were injected with rabbit serum (Sigma-Aldrich). The first injections of anti-ASGM1 or rabbit serum were performed 1 day before intranasal challenge with protease combined with OVA, and then every 5 days until sacrifice.

To test the role of NK cells in the resolution phase of CRS, mice were given an i.p. injection of 10 μl rabbit anti-ASGM1(Cedarlane Laboratories Ltd.) or rabbit serum (Sigma-Aldrich) after CRS development (protocol day 33) (Supplementary Fig. S13). The extent of inflammation was analyzed during resolution phase of CRS (protocol day 36).

Blood samples were taken by cardiac puncture, and differential cell counting was performed using the ADVIA 2120 Hematology System (Bayer HealthCare, Diagnostics Division, Tarrytown, NY). Tissue sections were stained with hematoxylin and eosin or Sirius red and analyzed by a pathologist in the Department of Pathology, Asan Medical Center, who was blind to the group assignments. Epithelial hyperplasia were scored on a scale of none (0), minimal (1), mild (2), moderate (3), to severe (4). Minimal was defined as barely detectable, mild as slightly detectable, moderate as easily detectable, and severe as very evident^48^. The maximal mucosal thickness was measured at the transition zone of the olfactory and respiratory epithelia using an image analysis system (cellSens Standard 1.7). Infiltration of eosinophils in the lamina propria was expressed as the number of cells/HPFs. Splenocytes from CRS mice were stimulated with 12-myristate 13-acetate (PMA; 20 ng/mL) and ionomycin (500 ng/mL) for 4 h. Lymphocytes were gated on FSC/SSC, and the CD107a expression of CD3ε-NKp46^+^ mouse NK cells was analyzed by flow cytometry. All experimental protocols were approved by the Institutional Animal Care and Use Committee of the Asan Institute for Life Science.

### Detection of apoptotic cells in CRS mice

To detect apoptotic eosinophils in sinonasal tissues of CRS mice, slides were first stained with Sirius red to identify eosinophils and then stained by the terminal deoxynucleotidyl transferase-mediated dUTP nick end-labeling (TUNEL) technique using *in situ* cell death detection kits (Roche Diagnostics, Laval, Quebec, Canada), according to the manufacturer’s instructions. Briefly, sections were incubated with 0.3% H_2_O_2_ in PBS for 5 min at room temperature for quenching of endogenous peroxidase activity, treated by microwave irradiation for 5 min in citrate buffer (0.1 M citrate buffer at pH 6.0) for antigen unmasking, and immersed for 30 min at room temperature in blocking buffer (0.1 M Tris-HCl at pH 7.5, containing 3% BSA and 20% bovine serum). Slides were then incubated for 1 h in a humidified chamber at 37 °C with the TUNEL reaction mixture containing terminal deoxynucleotidyl transferase and fluorescein-conjugated dUTP. For detection of fluorescein-labeled DNA fragments, slides were incubated with a sheep anti-fluorescein antibody conjugated to horseradish peroxidase for 30 min at 37 °C. The TUNEL signal was observed using the diaminobenzidine substrate kit (Roche Diagnostics). Thereafter, the sections were counterstained with Harris’ hematoxylin (Sigma-Aldrich). Apoptotic cells were identified as those cells with dark brown TUNEL-positive nuclei and showing the morphologic appearance of apoptosis, such as marked loss of cytoplasm and nuclear condensation^6^,^49^. Apoptotic eosinophils were identified as those cells with colocalization of TUNEL-positive nuclear staining and Sirius red-positive cytoplasmic staining. We evaluated the percentage of TUNEL-positive eosinophils relative to total eosinophils/HPF, which was determined with a light microscope at a magnification of 1000×^6^,^50^.

### Statistical analysis

All data were analyzed by using GraphPad Prism v.4.00 (GraphPad Software Inc.). Comparisons of paired values were performed using the nonparametric Wilcoxon matched-pairs signed-rank test. The groups were compared by using a nonparametric Mann-Whitney *U* test and Friedman test. Correlations were evaluated by Spearman’s correlation analysis. Statistical significance was defined as *P* < 0.05, and the degree of significance was presented as follows: *P < 0.05; **P < 0.01; ***P < 0.001.

## Additional Information

**How to cite this article**: Kim, J. H. *et al*. Natural killer cells regulate eosinophilic inflammation in chronic rhinosinusitis. *Sci. Rep*. **6**, 27615; doi: 10.1038/srep27615 (2016).

## Supplementary Material

Supplementary Information

## Figures and Tables

**Table 1:** Clinical characteristics of the CRS patients and control subjects.

Characteristics	CRS (n = 43)	Control (n = 18)
Age (y), median (range)	49 (20–72)	30 (25–40)
Gender (M:F)	35:8	5:13
Asthma (%)	7 (16.3)	0 (0)
Atopy (%)	7 (16.3)	0 (0)
Aspirin intolerance (%)	0 (0)	0 (0)
Blood eosinophil count (count/μl), median (range)	288 (22–840)	65 (20–170)

**Table 2:** Comparison of the CRS groups in terms of clinical features.

Parameter	No. (%) of patients or median (range)		*P*value
	Noneosinophilic CRS(n = 25)	Eosinophilic CRS(n = 18)	
Age, yr	43 (20–69)	56 (22–72)	0.021^*^
Sex, male	19 (76.0)	16 (88.9)	0.434^†^
Asthma	2 (8.0)	5 (27.8)	0.110^†^
Atopy	3 (12.0)	4 (22.2)	0.427^†^
NP	19 (76.0)	18 (100)	0.032^†^
Blood eosinophil absolute count, counts/μl	148 (71–779)	411 (22–840)	0.014^*^
Blood eosinophil percent,%	2.7 (1.0–10.5)	6.9 (0.2–12.0)	0.003^*^
Lund-Mackay CT score	12 (2–23)	12 (4–21)	0.569^*^

ECRS, eosinophilic chronic rhinosinusitis.

NP, nasal polyp.

^*^Mann-Whitney *U* test; ^†^Fisher’s exact test.
