# Supplementary material for: Natural killer cells regulate eosinophilic inflammation in chronic rhinosinusitis
Source: Sci Rep. 2016 Jun 8;6:27615. doi: 10.1038/srep27615 (PMC4897886; doi:10.1038/srep27615)
Supplement: Supplementary Information [file srep27615-s1.doc]

**Supplementary information**

**Natural killer cells regulate eosinophilic inflammation in chronic rhinosinusitis**

Ji Heui Kim, Go Eun Choi, Bong-Jae Lee, Seog Woon Kwon, Seung-Hyo Lee, Hun Sik Kim, and Yong Ju Jang


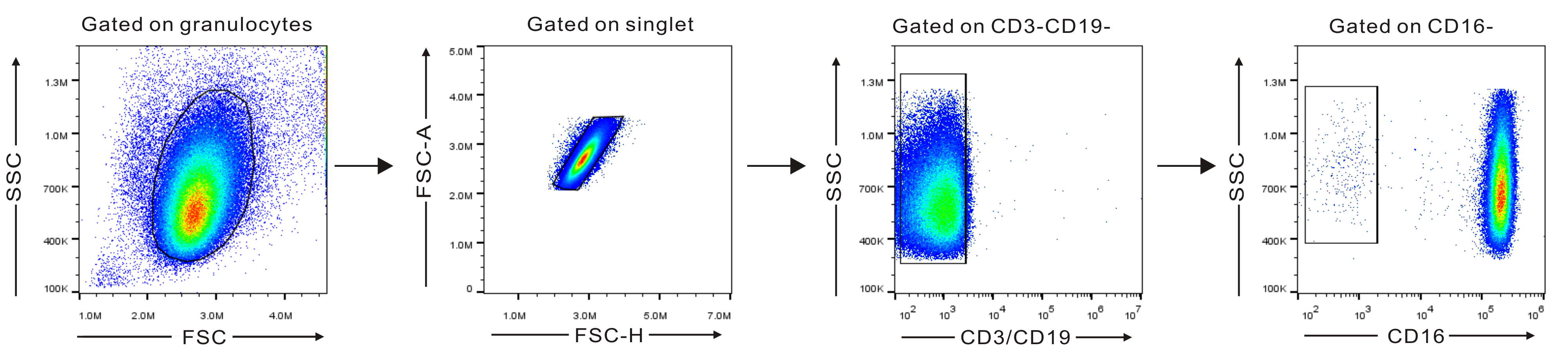


**Figure S1.** FACS gating strategy. Profiles showing the gating strategy for identifying the CD3-CD19-CD16- eosinophils within the granulocyte gate. Gating strategy: FSC vs. SSC, FSC-Height vs. FSC-Area, then CD3/CD19 vs. SSC, and CD16 vs. SSC.

**
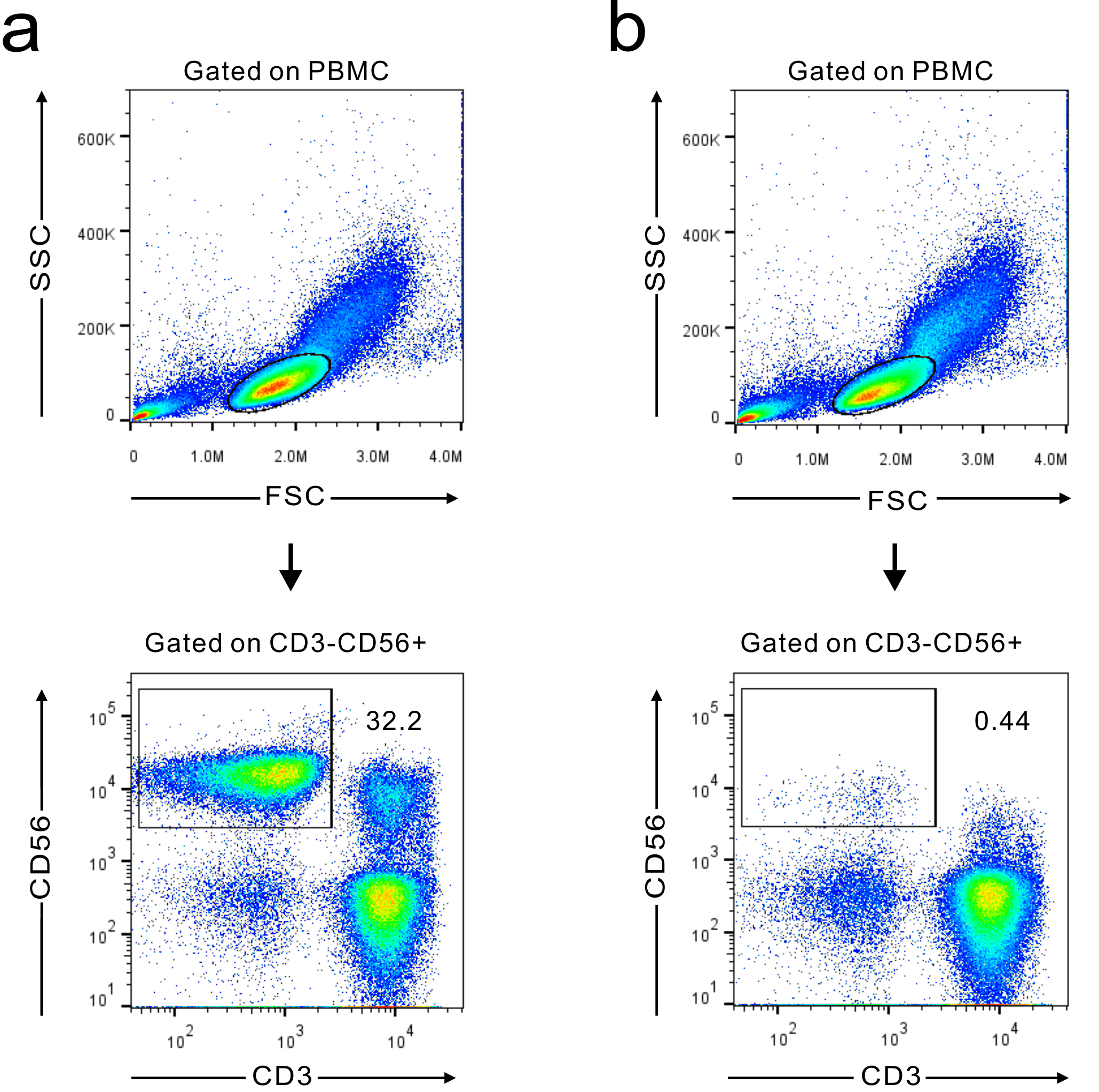
**

**Figure S2.** NK cell analysis in CD56-depleted lymphocytes. (**a**) Profiles showing the frequency of CD3-CD56+ NK cells within the lymphocyte gate. (**b**) CD56-depleted lymphocytes were obtained from PBMCs. The frequencies of NK cells in the depleted lymphocytes were <1%.

**
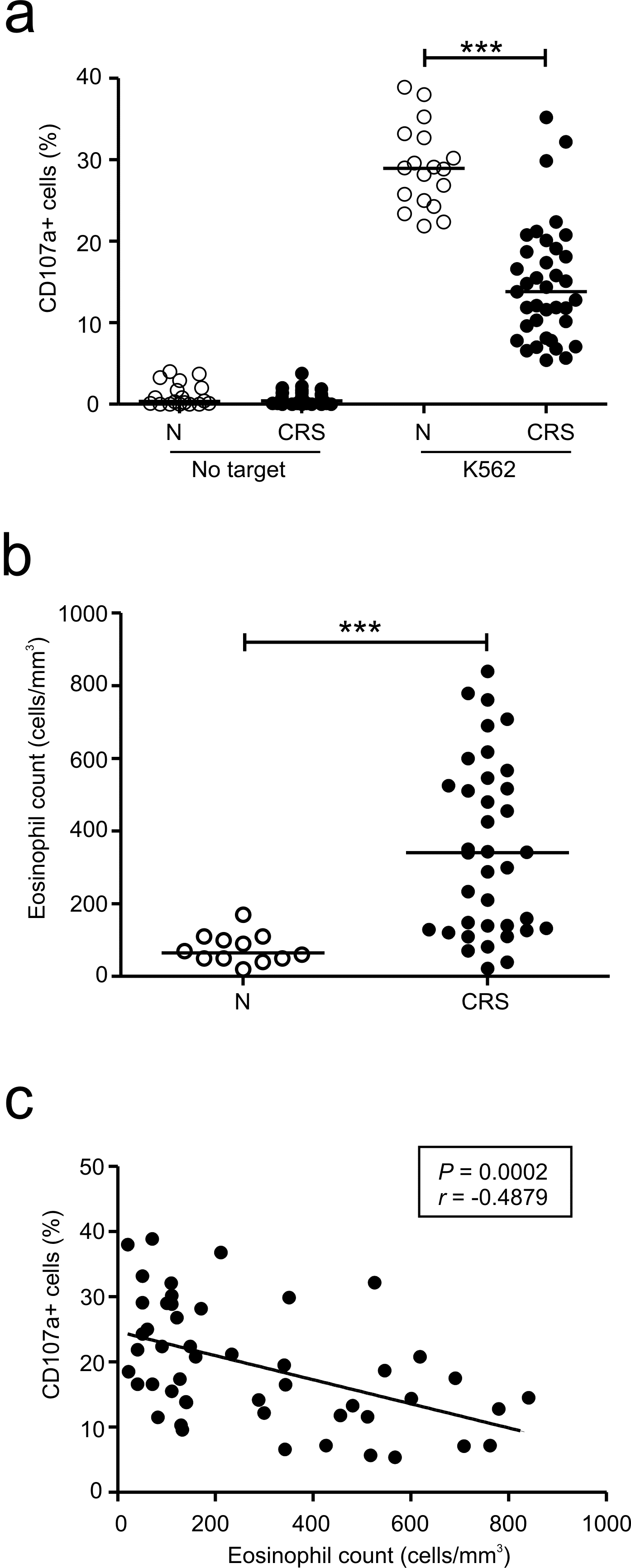
**

**Figure S3.** Peripheral blood eosinophil counts are associated with impaired NK cell function. (**a**) The degranulation of NK cells, as measured by cell surface expression of CD107a, after stimulation with K562 cells. Statistical dot plots showing the expression of CD107a by NK cells from the controls (N) (n = 18) or patients with CRS (n = 37) (**b**) Statistical dot plots showing the eosinophil counts in peripheral blood from the controls (N) (n = 12) or patients with CRS (n = 37). (**c**) The eosinophil counts correlated negatively with the percentages of CD107a-positive NK cells after stimulation with K562 cells. Horizontal bars denote the medians. ****P* < .001, Mann-Whitney *U* test (**a, b**) and Spearman correlation test (**c**).

**
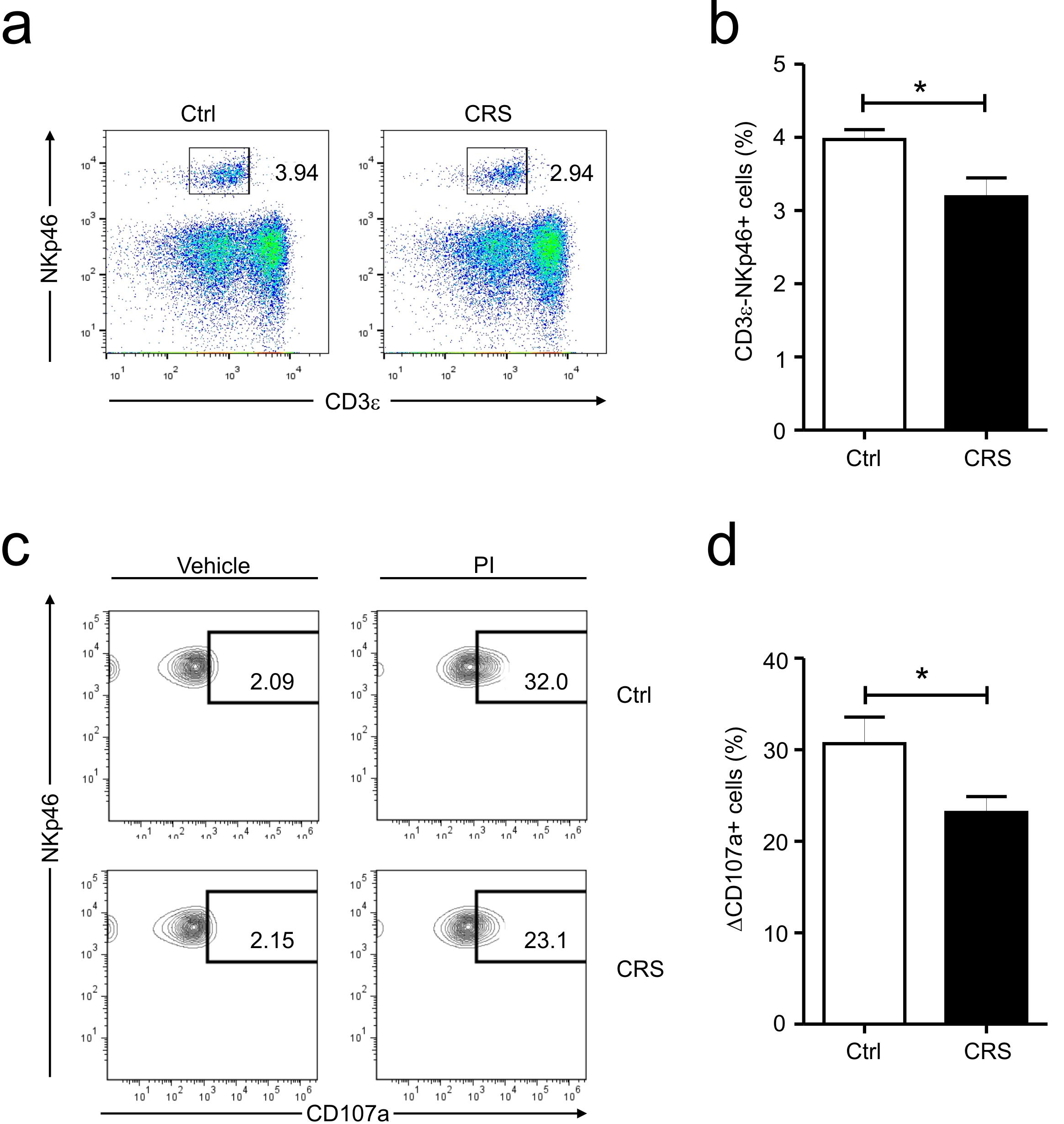
**

**Figure S4.** CRS mice have impaired NK cell function. (**a, b**) Spleen cells from control and CRS mice were isolated and stained for surface expression of CD3ε and NKp46. Representative FACS profiles (**a**) and statistical dot plots (**b**) showing the percentages of CD3ε-NKp46+ NK cells. (**c, d**) Splenocytes from control and CRS mice were treated with or without PMA and ionomycin. Representative FACS profiles (**c**) and statistical dot plots (**d**) showing the percentages of the surface expression of CD107a on CD3ε-NKp46+ NK cells. Data are expressed as means ± SDs. **P* < .05 by Mann-Whitney *U* test.

**
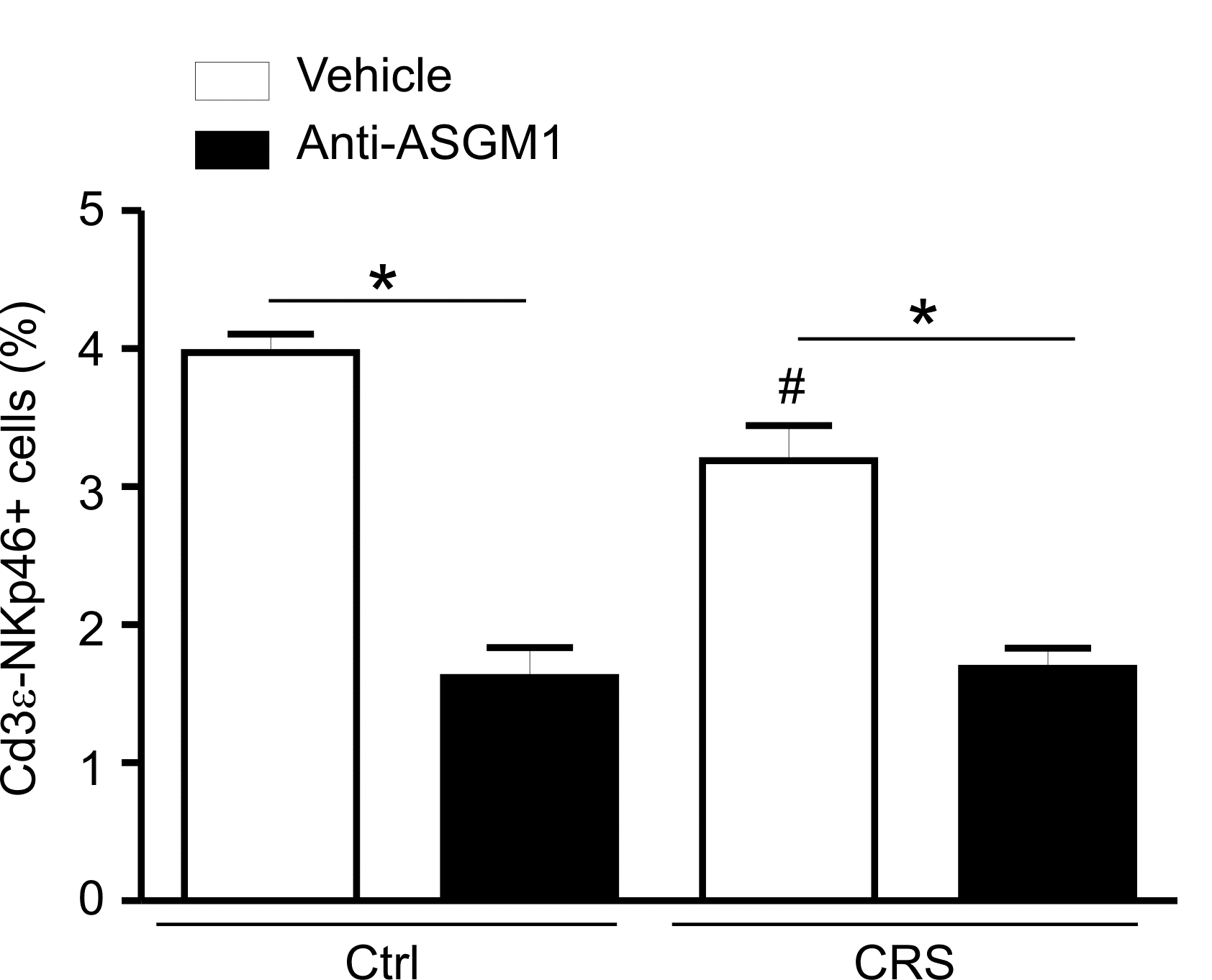
**

**Figure S5.** Anti-ASGM1 antibody depletes NK cells in CRS mice. Control and CRS mice were untreated or treated with i.p. injection of anti-ASGM1 antibody (10 μL) during the course of CRS development. The frequencies of NK cells (CD3ε-NKp46+) in the spleen of each group are shown. Data are expressed as means ± SDs. **P* < .05; ^#^*P* < .05 by Mann-Whitney *U* test.

**
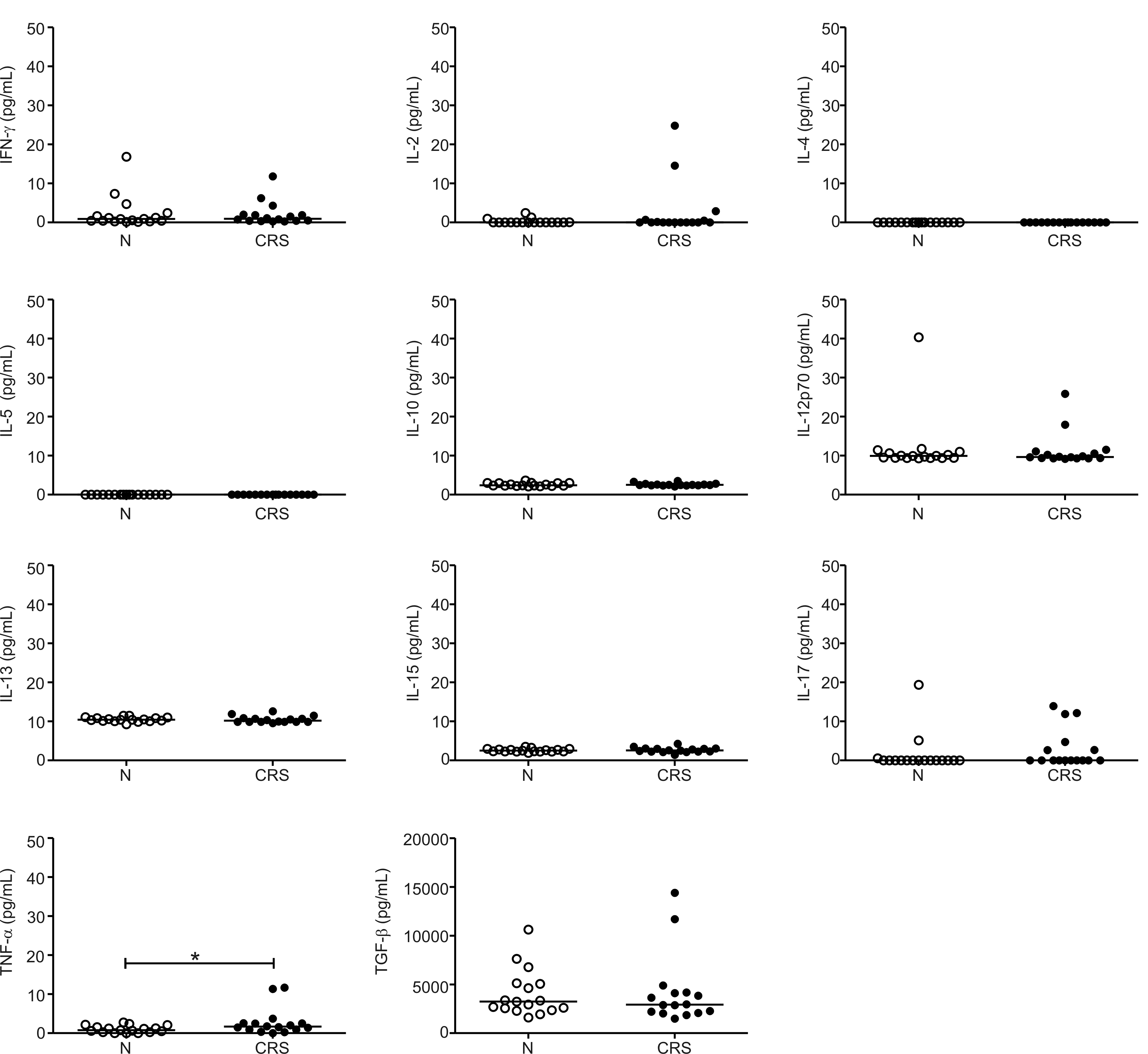
**

**Figure S6.** Comparison of the controls and CRS patients in terms of serum cytokine levels. The serum levels of the IFN-γ, IL-2, IL-4, IL-5, IL-10, IL-12p70, IL-13, IL-15, and IL-17 were measured by using Luminex multiplex assay. The TGF-β levels in the serum were assessed by using EIA. Statistical dot plots showing the levels of the indicated cytokines in the serum from the controls (N) (n=17) or patients with CRS (n=16). Horizontal bars denote the medians. **P*<0.05 by Mann-Whitney *U* test.

**
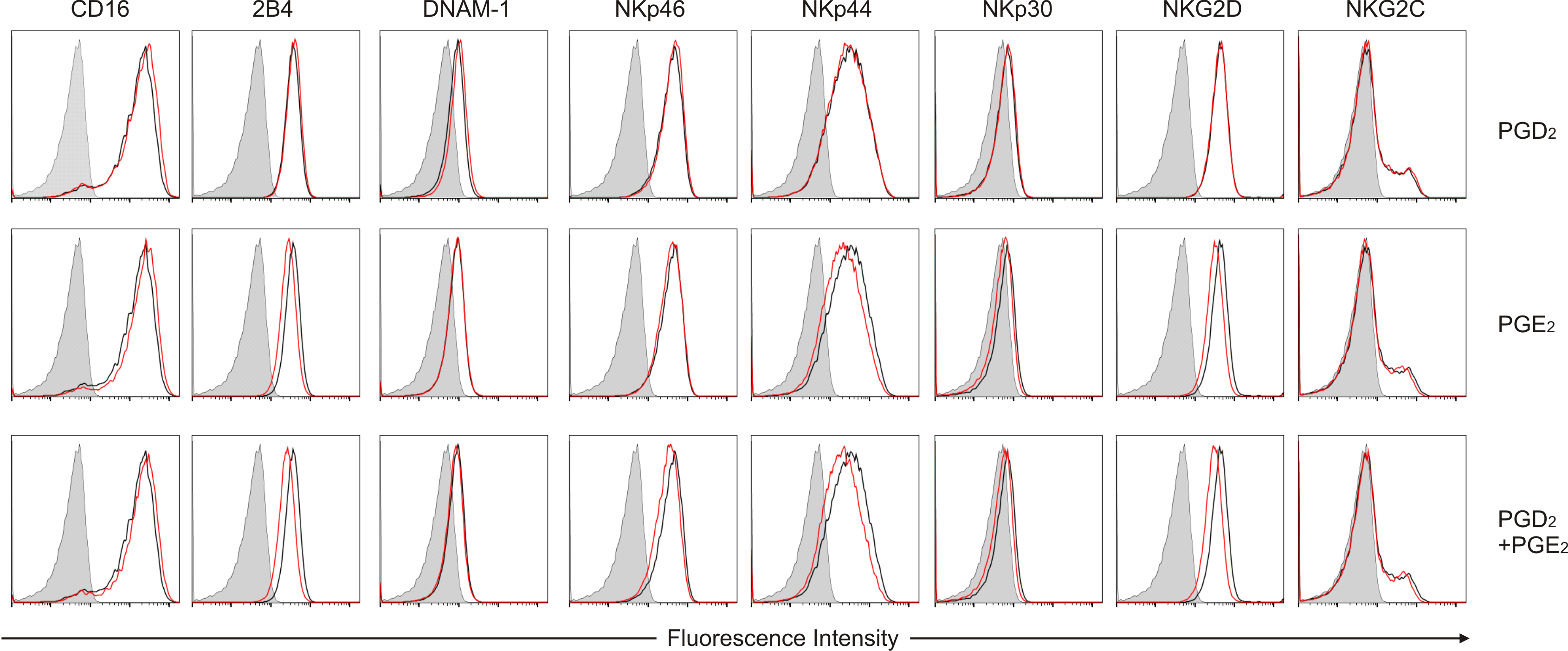
**

**Figure S7.** Effect of PGs on the expression of NK cell activating receptors. Representative FACS profiles showing the expression of the CD16, 2B4, DNAM-1, NKp46, NKp44, NKp30, NKG2D, and NKG2C receptors on gated NK cells after incubation with PGD_2_, PGE_2_, and a combination of PDG_2_ and PGE_2_ (red solid line), or with vehicle only (black solid line). Isotype control staining is shown as a shaded histogram.


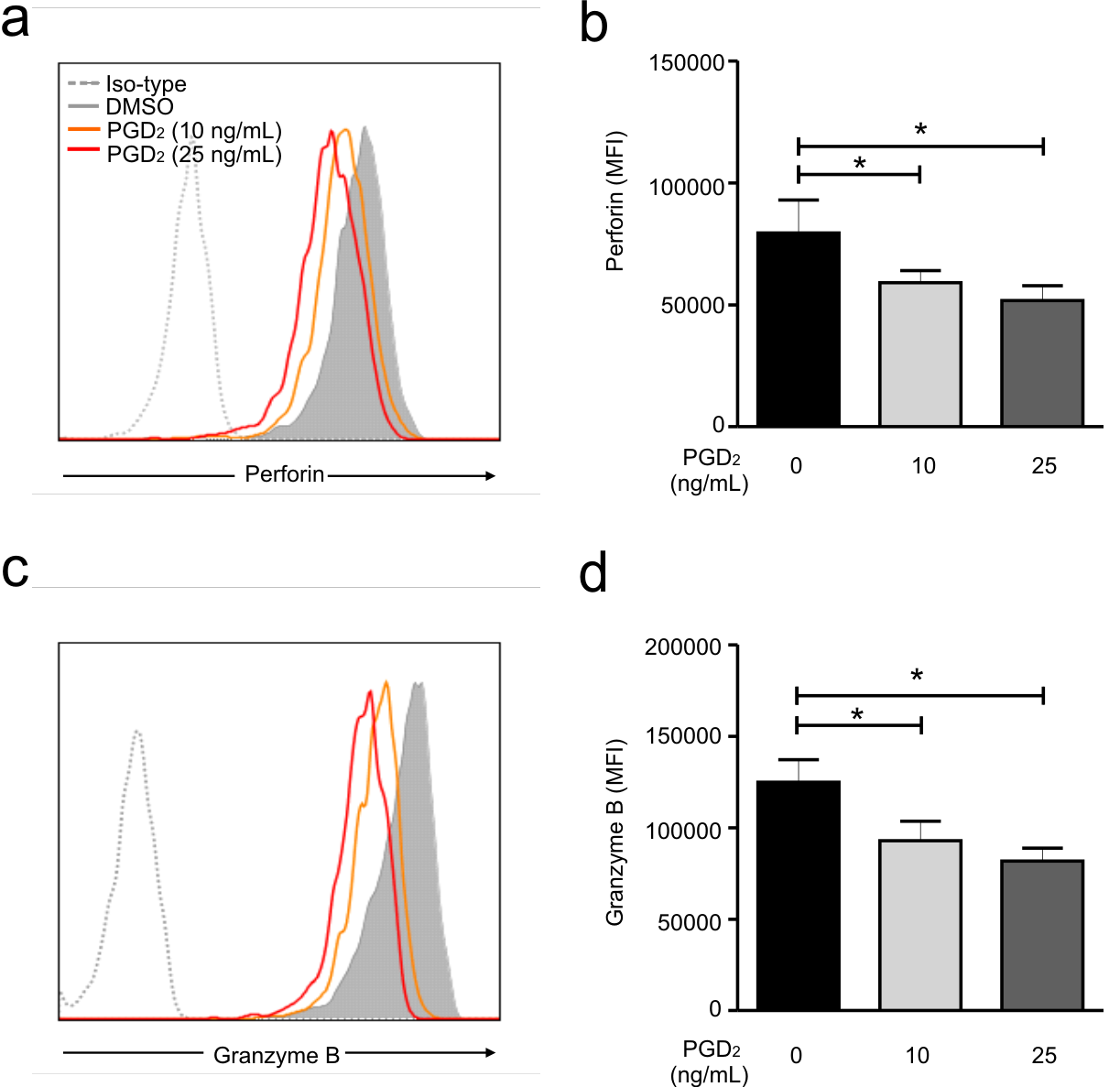


**Figure S8.** Effect of PGD_2_ on the expression of perforin and granzyme B in NK cells. (**a**, **c**) Representative FACS profiles showing the expression of the perforin (**a**) and granzyme B (**c**) in gated NK cells after incubation with the indicated concentrations of PGD_2_ for 5 h. (orange or red solid line), or with vehicle only (shaded histogram). Isotype control staining is shown as a grey dotted line. (**b, d**) Statistical bar charts showing the MFI of perforin (**b**) and granzyme B (**d**) in NK cells from the controls (n = 4). Data are expressed as means ± SDs. **P* < .05 by Mann-Whitney *U* test.

**
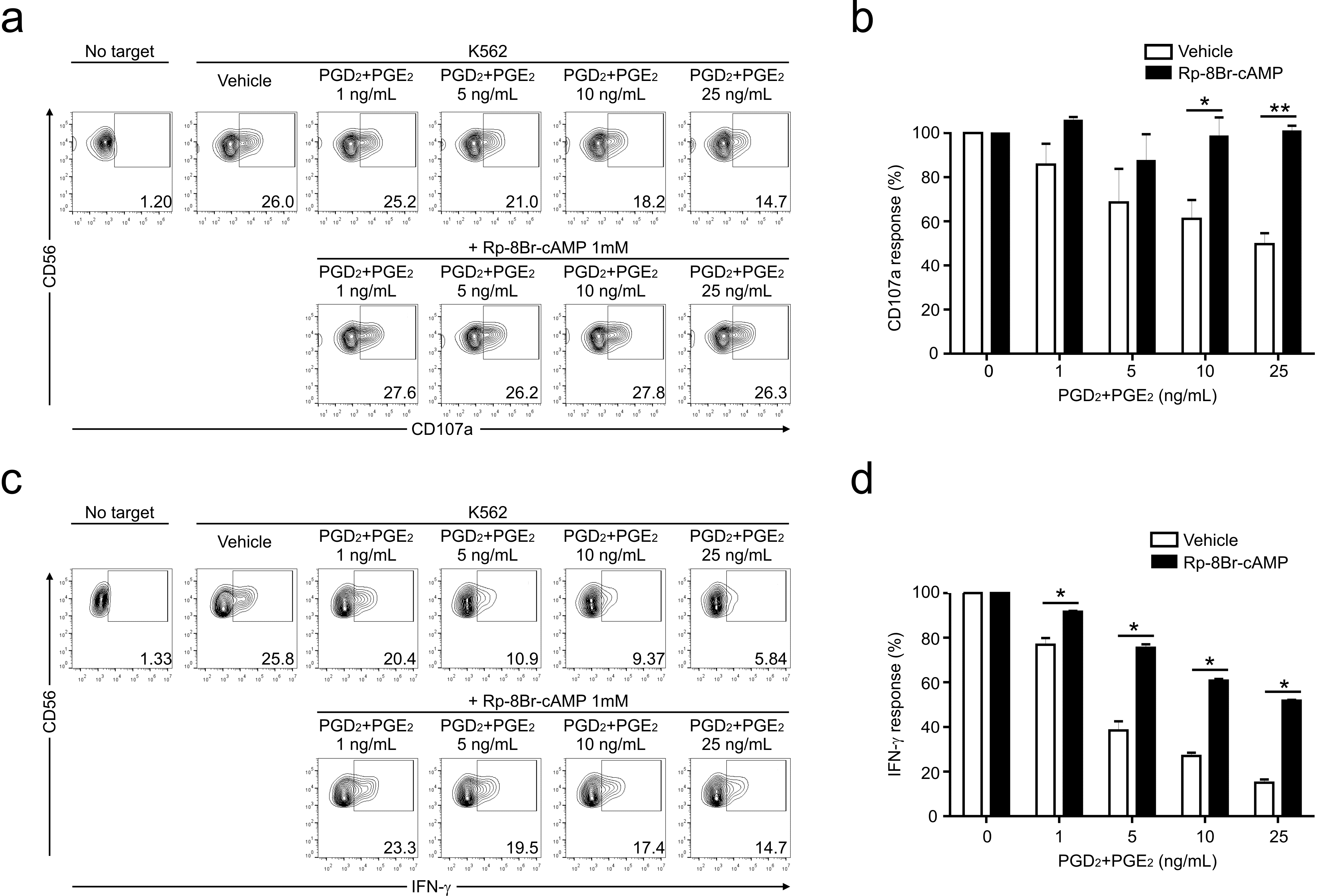
**

**Figure S9.** PG-induced NK cell dysfunction is prevented by Rp-8Br-cAMP. PBMCs were treated with the indicated concentrations of PGD_2_ and PGE_2_ in combination with Rp-8Br-cAMP (1 mM). Degranulation and intracellular IFN-γ expression of NK cells were assessed after incubation with K562. (**a, b**) Representative FACS profiles showing the percentages of CD107a+ NK cells (**a**) and IFN-γ+ NK cells (**c**). (**b, d**) Statistical bar charts showing the percent response of CD107a+ NK cells (**b**) and IFN-γ+ NK cells (**d**) with PGs relative to that with vehicle only in the presence or absence of Rp-8Br-cAMP. Data are expressed as means ± SDs. **P* < .05; ***P* < .01; ****P* < .001 by Mann-Whitney *U* test.

**
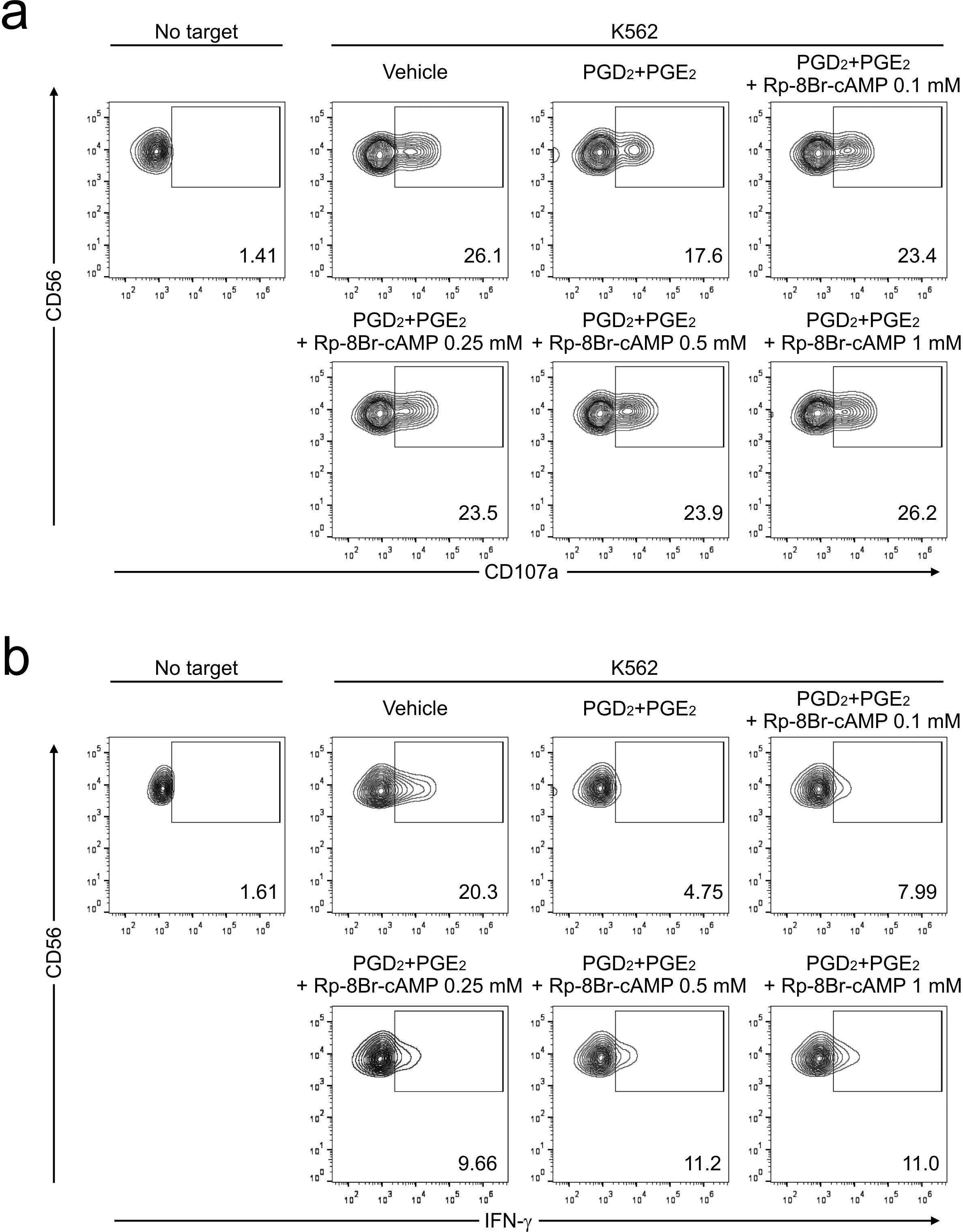
**

**Figure S10.** PG-induced NK cell dysfunction involves the cAMP pathway. PBMCs were treated with PGD_2_ and PGE_2_ (25 ng/mL) in combination with the indicated concentrations of Rp-8Br-cAMP. Degranulation and intracellular IFN-γ expression of NK cells was assessed after incubation with K562 cells. (**a, b**) Representative FACS profiles showing the percentages of CD107a+ NK cells (**a**) and IFN-γ+ NK cells (**b**).


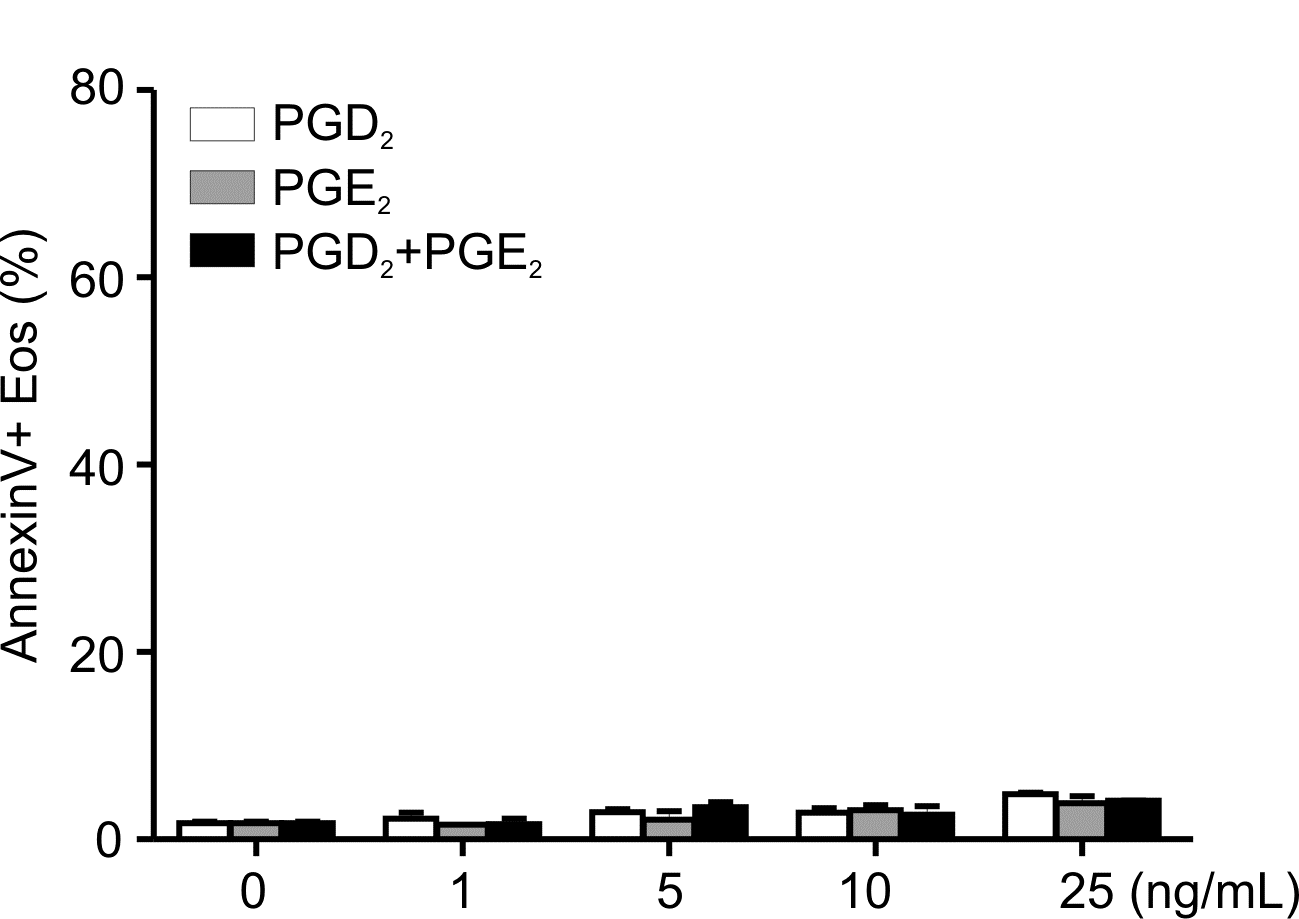


**Figure S11.** PGs per se do not affect eosinophil apoptosis during the time frame of the assay. Peripheral blood granulocytes from the patients with CRS were treated with PGD_2_, PGE_2_, or a combination of PGD_2_ and PGE_2_ for 4 hours as indicated. Following treatment, cells were analyzed by flow cytometry after staining with annexin V-FITC and with 7-AAD. Statistical bar charts showing the percentage of annexin V+ eosinophils. Data are expressed as means ± SDs.


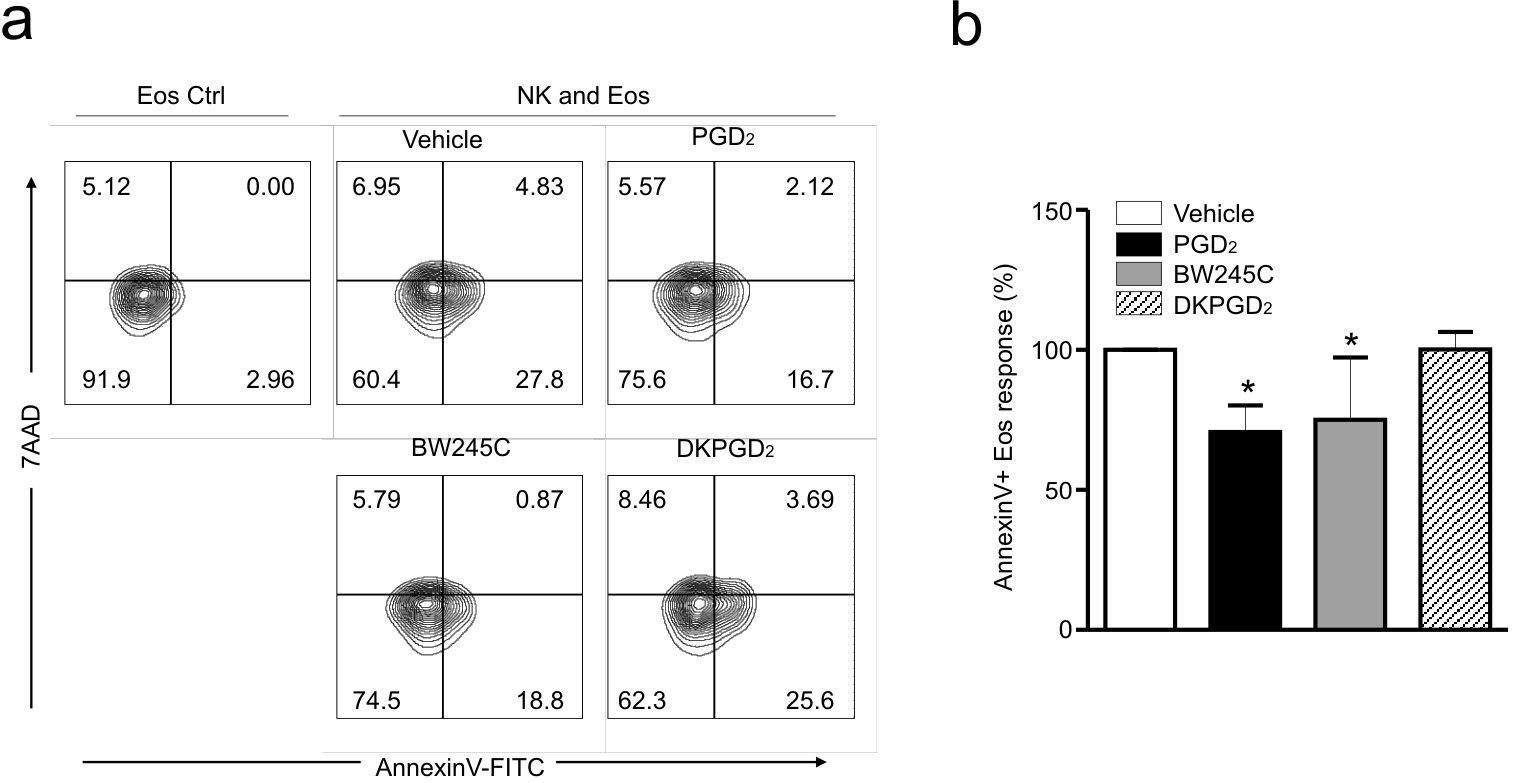


**Figure S12.** PGD_2_ inhibits NK cell-mediated eosinophil apoptosis though DP1. PBMCs were pretreated with PGD_2_ (25 ng/mL), BW245C (100 nM), or DKPGD2 (100 nM) and then incubated with autologous granulocytes. (**a**) Representative FACS profiles showing the effect of PGD_2_ receptor agonists on the percentage of annexin V+ eosinophils. (**b**) Statistical bar graphs showing the percent response of annexin V+ eosinophils to PGD_2_ receptor agonists relative to that with vehicle only. Data are expressed as means ± SDs. **P* < 0.05 by Mann-Whitney *U* test.


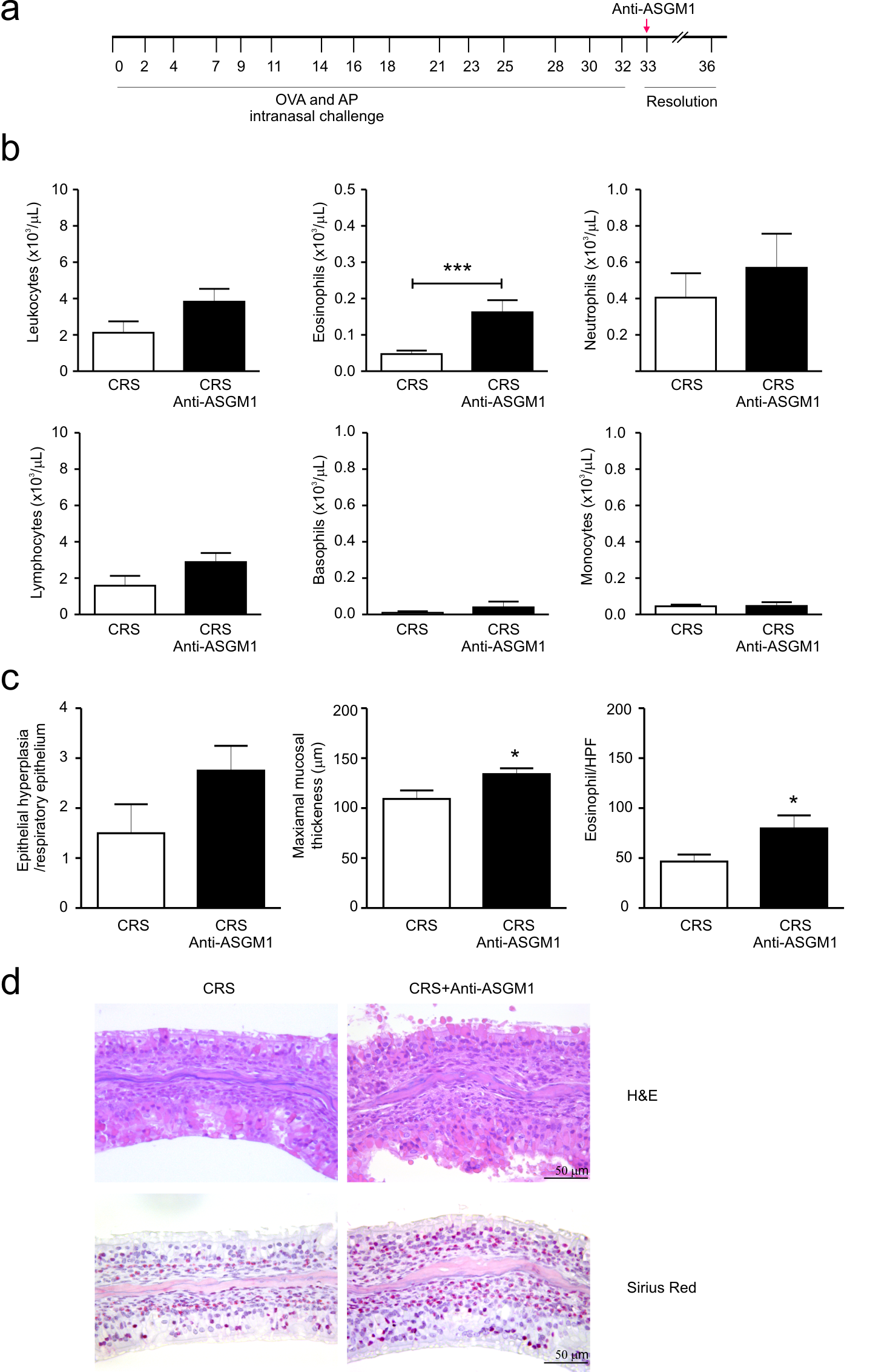


**Figure S13.** NK cell depletion after CRS development delays the resolution of eosinophilic inflammation in a murine model of CRS. (**a**) Schematic diagram showing the protocol used to deplete NK cells after CRS development. (**b**) Counts of leukocytes, eosinophils, neutrophils, lymphocyte, basophils, and monocytes in blood from each group of mice (n = 4). (**c**) Scores of epithelial hyperplasia (*left*), maximal mucosal thickness (*middle*) in hematoxylin and eosin-stained tissue sections, and eosinophil counts of the lamina propria (*right*) in Sirius red-stained tissue sections. (**d**) Representative photographs of hematoxylin and eosin (*upper*, original magnification ×400) and Sirius red (*lower*, original magnification ×400)-stained sections. Scale bars represent 50 μm. Data are expressed as means ± SDs. **P* < .05; ^#^*P* < .05, Mann-Whitney *U* test (**b, c)**.
